# Avian species functional diversity and habitat use: The role of forest structural attributes and tree diversity in the Midlands Mistbelt Forests of KwaZulu‐Natal, South Africa

**DOI:** 10.1002/ece3.10439

**Published:** 2023-08-31

**Authors:** Nasiphi Bitani, Craig P. Cordier, David A. Ehlers Smith, Yvette C. Ehlers Smith, Colleen T. Downs

**Affiliations:** ^1^ Centre for Functional Biodiversity, School of Life Sciences University of KwaZulu‐Natal Pietermaritzburg South Africa; ^2^ Ezemvelo KwaZulu‐Natal Wildlife Pietermaritzburg South Africa

**Keywords:** bird species community structure, forest conservation, forest structure, Mistbelt Forests

## Abstract

Forest transformation has major impacts on biodiversity and ecosystem functioning. Identifying the influence of forest habitat structure and composition on avian functional communities is important for conserving and managing forest systems. This study investigated the effect of forest structure and composition characteristics on bird species community structure, habitat use and functional diversity in 14 Mistbelt Forest patches of the Midlands of KwaZulu‐Natal in South Africa. We surveyed bird communities using point counts. We quantified bird functional diversity for each forest patch using three diversity indices: functional richness, functional evenness and functional divergence. We further assessed species‐specific responses by focussing on three avian forest specialists, orange ground‐thrush *Geokichla gurneyi*, forest canary *Crithagra scotops* and Cape parrot *Poicephalus robustus*. We found that bird community and forest‐specialist species responses to forest structure and tree species diversity differed. Also, forest structural complexity, canopy cover and tree species richness were the main forest characteristics better at explaining microhabitat influence on bird functional diversity. Forest patches with relatively high structural complexity and tree species richness had higher functional richness. Different structural characteristics influenced habitat use by the three forest specialists. Tree species diversity influenced *C. scotops* and *G*. *gurneyi* positively, while *P. robustus* responded negatively to forest patches with high tree species richness. Our study showed that site‐scale forest structure and composition characteristics are important for bird species richness and functional richness. Forest patches with high tree species diversity and structural complexity should be maintained to conserve forest specialists, bird species richness and functional richness.

## Graphical abstract

We investigated the effect of forest structure and composition characteristics on bird species community structure, habitat use and functional diversity in 14 Mistbelt Forest patches of the Midlands of KwaZulu‐Natal, South Africa. We found that bird community and forest‐specialist species responses to forest structure and tree species diversity differed. Our study showed that site‐scale forest structure and composition characteristics are important for bird species richness and functional richness.

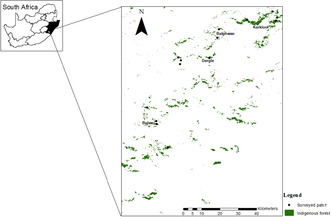

## 1 INTRODUCTION

Land use change is the most prominent threat to forest systems impacting biodiversity and ecosystem functioning globally (Aquilué et al., 2020; Bregman et al., 2016; Morelli et al., 2020). The transformation of natural habitats leads to changes in forest structure, composition and function, reducing native biodiversity and affecting different taxa's functional diversity, including birds (Ehlers Smith et al., 2017a; Foley et al., 2005; Newbold et al., 2020). These effects have driven a conservation issue and a lot of ecological questions relating to the impact of forest vegetation structural characteristics on species diversity. There is a strong link between forest structure and composition with habitat heterogeneity and ecosystem functioning (Rodrigues et al., 2017; Vanbergen et al., 2007). Diverse forest structures and composition provide many functions, including strengthening species community stability (Ehbrecht et al., 2017; Sanderson et al., 2002), microhabitat formation and supporting species with specialised niches (Brockerhoff et al., 2017; Dekeukeleire et al., 2019). Avian communities are key in the ecosystems and contribute to ecosystem services and maintain ecosystem functioning (Bregman et al., 2016; Benedetti et al., 2020). Depending on their specialisation, bird species provide ecosystem services such as seed dispersal (Egerer et al., 2018), pollination (Clout & Hay, 1989) and pest control (Kellermann et al., 2008). Birds are an important taxon to study the influence of forest structure and composition because they are habitat quality indicators and respond relatively quickly to environmental change (Boesing et al., 2018; Cosset et al., 2020). In the last decade, most studies investigating the effects of either forest structure or composition on bird species diversity at a landscape level have mainly focussed on the functional biodiversity aspect (Benedetti et al., 2020; Broms et al., 2016; Bueno et al., 2018; Burivalova et al., 2015; Ehlers Smith et al., 2017a, 2018; Gumede, Ehlers Smith, Ngcobo, et al., 2022; Ngcobo et al., 2022). This approach has been driven by conservation strategies increasingly targeting maintaining ecological functions (Mace, 2014). This is important because species' functional groups indicate the resilience of ecosystems and maintain ecosystem functions and processes (Alexander et al., 2019; Bregman et al., 2016; Flynn et al., 2009). Also, this approach explains the potential effects of habitat change on the functioning of ecosystems (De Coster et al., 2015; Morante‐filho & Morante‐filho, 2021). However, only focussing on certain functional groups or using only community measures can conceal species‐specific responses to habitat variation (Lemaître et al., 2012). Different species use habitats differently, and bird species' responses to habitat structural changes are species‐specific (Berl et al., 2015; Bouvet et al., 2016; Ehlers Smith et al., 2017b; Gumede, Ehlers Smith, Ehlers Smith, et al., 2022). For example, bird species with specialised niches that are functionally distinct are more vulnerable than others (Flynn et al., 2009). Therefore, exploring species‐level responses to forest structure and composition is essential, especially for forest specialists more vulnerable to habitat changes.

Forest ecosystems are three‐dimensional, with different characteristics influencing bird species' habitat use (Thiollay, 1992). A recent study using camera traps to assess species habitat use showed bird species‐specific vertical stratification patterns in temperate forests (Godoy‐Güinao et al., 2023). Among others, bird habitat selection in forest systems is influenced by the structural features of forests like deadwood, canopy cover, canopy openness (Bradfer‐Lawrence et al., 2020) and vertical foliage cover (grass, shrub, herbaceous, tree) and height (Gumede, Ehlers Smith, Ngcobo, et al., 2022; Maseko et al., 2019). Several studies have linked habitat selection and use to habitat quality and resource availability (Anderson et al., 1979; Böhm & Kalko, 2009; Thiel et al., 2021). For example, the availability of certain features and resources (nest sites, food availability) varies from the ground to the forest canopy. Considering the vertical stratification in bird species use in forests, it is important to understand habitat use in relation to habitat structural characteristics.

The indigenous forest biome is the smallest in South Africa, estimated to be 0.41% (Deng et al., 2020) but of disproportionately high conservation and ecological value (Downs & Symes, 2004; Lawes et al., 2000). Mismanaged fires, timber harvesting and transformation for plantations are the main threats to the Southern Mistbelt Forests (Mucina et al., 2006). These forests in KwaZulu‐Natal were generally heavily logged for approximately 150 years, and the exploitation was dominant between the 1800s and early 1940s when illegal logging was prohibited (King, 1941; Wirminghaus et al., 1999). Selective logging affected the ecology of these forests, and the system has been left to recover naturally through successional processes (Adie et al., 2013). Government agencies or private owners have prioritised the forest biome for management. Despite being protected, the Mistbelt Forests remain illegally harvested (Grieve & Downs, 2015). Approximately 6.5% of avian species in South Africa are classified as forest‐depend (Oatley, 1989), and about half are experiencing range declines with >16% average range decline since 1992 (Cooper et al., 2017). An understanding of forest features influencing habitat use to allow for the long‐term persistence of birds, particularly forest specialists in these forests, is essential.

Understanding bird species' habitat use and selection allows for sustaining essential forest structural characteristics defining bird species microhabitats to prevent species loss (Díaz et al., 2005) and developing integrated habitat management plans (Hewson et al., 2011). The Southern Mistbelt Forests are multilayered, naturally fragmented patches nested within a grassland mosaic landscape (Mucina et al., 2006). Human activities have further aggravated fragmentation with species' structure, configuration and composition changes within the system (Adie et al., 2013; Leaver et al., 2019). Therefore, the Southern Mistbelt Forest is a good system to study the influence of forest structure on forest bird specialists. In South African forests, the range of the selected bird species has declined substantially because of habitat destruction (Cooper et al., 2017). As habitat specialists, the selected bird species are more vulnerable to habit disturbance, hence are valuable as focal species in assessing their habitat preference. In this study, we selected three forest‐dependent bird species listed in Oatley (1989) as focal species for species‐specific responses. The orange ground‐thrush (*Geokichla gurneyi*), forest canary (*Crithagra scotops*) and Cape parrot (*Poicephalus robustus*) are forest specialist species that differ in their vertical use of the forest strata (Hockey et al., 2005). The orange ground‐thrush is a forest ground‐dwelling bird and also feeds mostly on the ground; vegetation composition is essential for this species (Gumede, Ehlers Smith, Ehlers Smith, et al., 2022); the forest canary feeds mainly on seeds in the forest understory, while the Cape parrot is rather a forest canopy hence the importance of vertical structural complexity to maintain the populations of this species. The average range decline of the Cape parrot, forest canary and orange ground‐thrush is 54%, 10% and 8%, respectively, in South African forests (Cooper et al., 2017). If habitat destruction continues, it will significantly impact the remaining bird species population. Consequently, their presence and habitat preference can be used to indicate the forest patch habitat quality (Gumede, Ehlers Smith, Ehlers Smith, et al., 2022; Gumede, Ehlers Smith, Ngcobo, et al., 2022).

Previous studies assessing avian communities in the KwaZulu‐Natal Midlands Mistbelt Forest patches have either focussed on certain landscape characteristics (e.g. Wethered & Lawes, 2003, 2005) or specific structural characteristics (snags) (Downs & Symes, 2004) or seasonality differences (Symes et al., 2002). This study fills an important knowledge gap on the responses of bird communities and specific forest‐dependent species on vegetation structure and composition characteristics. Our study examined the effects of the forest structure and composition on the avian community structure of the KwaZulu‐Natal Midlands Mistbelt Forests in South Africa. Specifically, we asked how the forests' structural and compositional characteristics affect the bird species' community structure, richness and functional diversity. We then selected three forest specialists (orange ground‐thrush, forest canary and Cape parrot) occupying different vertical forest profile strata (i.e. ground‐dwelling, mid‐story, canopy) to assess the species‐specific responses. Focussing on these forest specialists, we explored how forest structural and compositional characteristics influenced forest bird specialists' habitat use and preference. We predicted that increased structural complexity would increase bird species richness and functional diversity. Forest specialist bird species have specific microhabitat requirements (Murphy et al., 2018) with a strong vertical stratification in habitat use (Godoy‐Güinao et al., 2023). Also, the selected focal forest specialist species differ in their forest strata habitat use. Given these known differences in forest habitat selection (see Study species), we hypothesised that the forest structural characteristics would affect species‐specific responses for the selected avian forest specialists differently. For example, we expected important forest structural characteristics for the ground‐dwelling orange ground‐thrush would be leaf litter, herbaceous cover and grass. Also, we expected saplings and herbaceous cover to be an essential structural characteristic for the understory forest canary, while for the Cape parrot, canopy closure or openness would be important.

## 2 METHODS AND MATERIALS

### 2.1 Study species

The orange ground‐thrush is a near‐threatened uncommon, ground‐dwelling forest specialist bird species (Hockey et al., 2005). It is insectivorous, feeding mainly on earthworms and insects (Hockey et al., 2005). It nests in a bowl‐shaped cup, avoiding dense foliage about 1–2 m above the forest floor (Tarboton, 2001; Tarboton & Roberts, 2011). The forest canary is a small (15 g), granivorous forest understory bird (Hockey et al., 2005). This species is nearly endemic to South Africa, with a marginal distribution in Swaziland and Lesotho (Ward et al., 2003). The forest canary is sedentary and flocks during the nonbreeding season (Hockey et al., 2005), and nests in a cup in a forest tree or sapling between 1 and 6 m (Tarboton, 2001; Tarboton & Roberts, 2011). The Cape parrot is a relatively large (300 g) endangered, rare forest specialist endemic to South Africa. The estimated population is <2000 birds (Downs et al., 2014). Some main threats to the Cape parrot are habit destruction, fragmentation and specific feeding and nesting requirements (Downs et al., 2014; Leaver et al., 2022; Symes & Downs, 2002; Wirminghaus et al., 1999, 2001, 2002). It mainly feeds on the endocarp (kernels) of *Afrocarpus* or *Podocarpus* spp. fruits and then those of other indigenous forest fruits when these are unavailable, or they feed on exotics outside of forests (Hockey et al., 2005; Wimberger et al., 2023; Wirminghaus et al., 2002). It generally nests in a secondary cavity in a yellowwood tree, often 15 m above the ground (Tarboton, 2001; Wirminghaus et al., 2001). Usually, the same cavity is used in successive years (Hockey et al., 2005; Tarboton, 2001; Wirminghaus et al., 2001).

### 2.2 Study area

**FIGURE 1:**
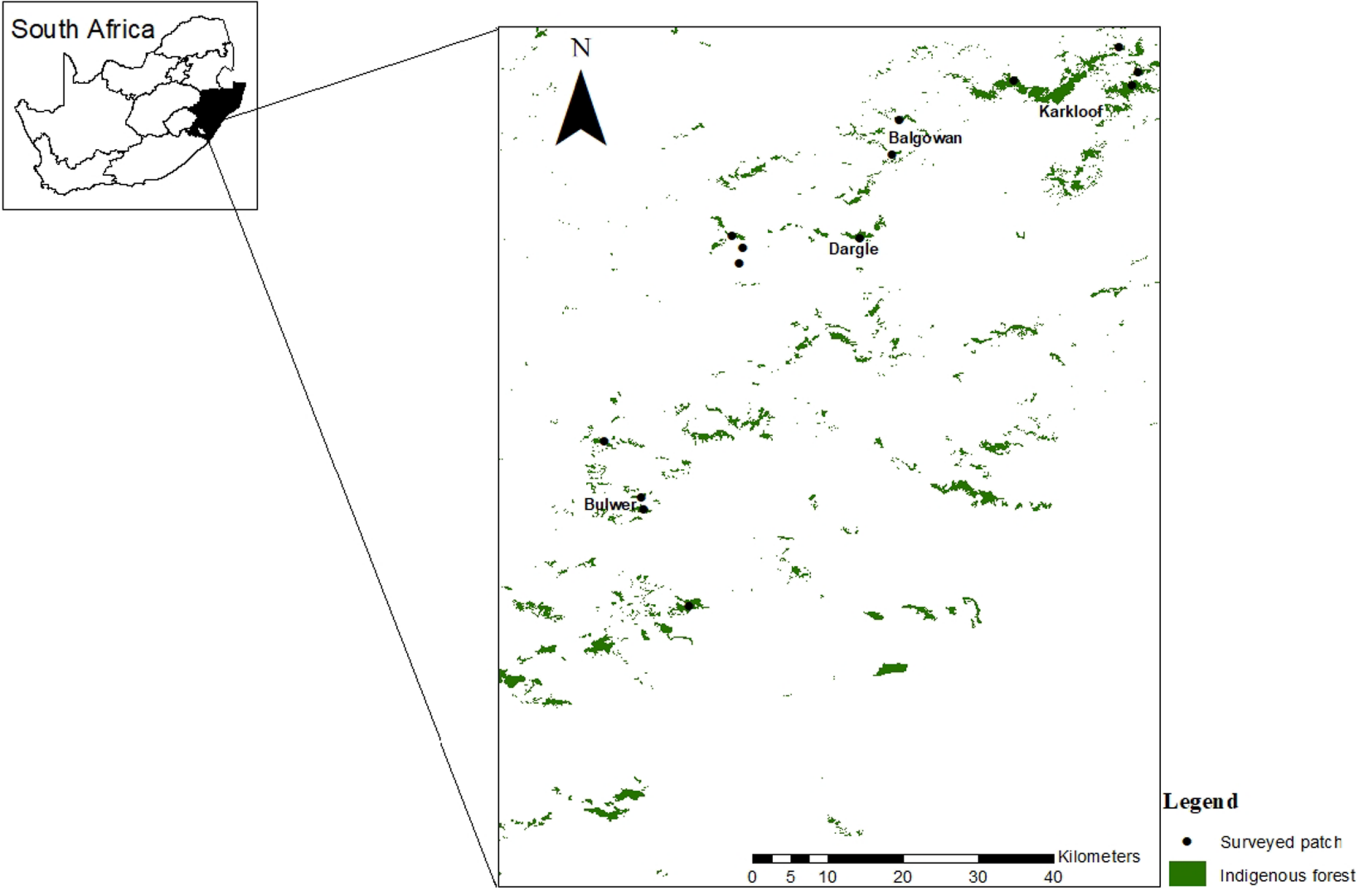
Distribution of 14 Mistbelt forest patches of the Midlands of KwaZulu‐Natal Province, South Africa, used in this study. (Black dots represent surveyed forest patches).

Our study was conducted from October 2020 to September 2021 in 14 selected patches of varying sizes (2.2–1685 ha) in four regions within the Midlands indigenous forests of KwaZulu‐Natal Province, South Africa (Table 1, Figure 1). The four different regions studied were: Karkloof (*n* = 4), Dargle (*n* = 4), Balgowan (*n* = 2), and Bulwer (*n* = 4), all categorised as Natal Midlands Mistbelt Forests (Table 1, Figure 1). The studied regions receive summer rainfall with frequent summer mist and have similar altitudes, average rainfall and temperature (Downs & Symes, 2004; Kotze & Samways, 1999). The Mistbelt mixed *Afrocarpus*/*Podocarpus* forests in the midlands of KwaZulu‐Natal occur on steep south‐facing slopes comprised of a few large patches (>1650 ha) surrounded by small patches (Lawes et al., 2004). The landscape surrounding the Mistbelt forest patches is mainly grassland and exotic forestry plantations (Leaver et al., 2022).

**TABLE 1:** Studied Mistbelt forest patches in the KwaZulu‐Natal Midlands, South Africa.

Region	Forest patch	Coordinates	Patch size (ha)	Point counts (*n*) per season	Dominant surrounding matrix
Karkloof	Karkloof of Nature Reserve^a^	29°17′50″ S; 30°13′59″ E	1685	98	Grassland
	Benvie Farm^a^	29°18′08″ S; 30°22′26″ E	101	17	Commercial plantation
	Mbona Private Nature Reserve^a^	29°15′27″ S; 30°21′29″ E	679	66	Commercial plantation
	L'Abri^a^	29°17′08″ S; 30°23′40″ E	199	19	Commercial plantation
Dargle	Maritzdaal Forest^a^	29°29′05″ S; 30°02′57″ E	558	46	Grassland
	Wakefield Forest^a^	29°29′06″ S; 29°54′28″ E	4.5	3	Grassland
	Waterfall Forest^a^	29°30′54″ S; 29°54′18″ E	2.2	1	Grassland
	Sharedown (New Forest)^a^	29°28′57″ S; 29°53′44″ E	112	17	Grassland
Balgowan	Milestone Forest Walk^a^	29°22′57″ S; 30°05′40″ E	77	6	Commercial plantation
	Rameron (Boshoek complex)^a^	29°20′34″ S; 30°05′43″ E	207	15	Grassland
Bulwer	Ingelabantwana Nature Reserve^b^	29°43′43″ S; 29°44′35″ E	338	24	Grassland
	Xotsheyake Nature Reserve^b^	29°47′46″ S; 29°47′16″ E	98	10	Commercial plantation
	Marutswa Nature Reserve^b^	29°48′36″ S; 29°47′25″ E	268	22	Commercial plantation
	Nxumeni (Nkwezela State Forest)^b^	29°55′38″ S; 29°50′42″ E	385	27	Commercial plantation

*Note*: Superscripts indicate landownership with privately (^a^) owned and state‐owned (^b^) forest patches.

### 2.3 Bird species survey

As per Ehlers Smith et al. (2017a) and Maseko et al. (2019), in ArcGIS 10.6 (ESRI, Redlands, CA, USA), grids of 200 × 200 m were overlaid using the recent indigenous forest layer (South African National Land‐Cover 2018) for the selected 14 patches. The grid axes (i.e. the grid lines crossing points) were assigned a survey point 200 m apart in all surveyed patches. We had 371 fixed‐radius point counts (Table 1) per season within the 14 selected forest patches, and the number of points per patch varied depending on the patch area (ha). We then projected the point counts into a global position system (GPS), then using a Garmin eTrex 10 (USA), we located these in the field. If the points were inaccessible in the field, we created a new point, ensuring it was within 50 m of the projected point. We used a fixed radius point count to identify bird species and recorded all bird species visible or audible within a 100 m radius. We recorded the number of individuals per identified bird species at each bird survey point. The point‐count surveys were conducted for 3 h from sunrise, sampling each survey point for 10 min and the number of points for each day varied depending on the openness of the forest patch. We did not record the bird species if we were unsure of the species' identification (*n* = 1). Each point count was surveyed once in each season (i.e. breeding from October 2020 to March 2021; the nonbreeding from April 2021 to September 2021). We then created a matrix of functional traits (Table S1) that captured bird functional roles in ecosystems and response to disturbance (Ehlers Smith et al., 2018; Flynn et al., 2009). The matrix included bird nesting type (i.e. ball/cup, cavity, or platform), body mass, primary diet (i.e. carnivore, frugivory, omnivory, nectarivory, granivory or insectivory), foraging strategy (i.e. harvest, terrestrial probe, arboreal probe, glean, hawk, perch and swoop or various) and habitat specificity (i.e. specialist or generalist) (Ehlers Smith et al., 2018; Flynn et al., 2009; Hockey et al., 2005). Species richness and abundance were calculated per surveyed point as the cumulative species number and number of individuals, respectively. Then, both were pooled as the total number of species and relative species abundance per patch (Ehlers Smith et al., 2017a).

### 2.4 Forest structure and composition sampling

Four‐quarters were established around each bird point survey to measure habitat structure and composition within a 20 m radius. We ran two tape measures diagonally to create the four quarters totalling 100%. Within each quarter, we visually estimated the percentage of understory cover for bare ground, leaf litter, grass, herbaceous plants, woody plant (saplings ≤2 m) and water. We also recorded the average height of grass, herbaceous and woody plants and the maximum tree height. We counted the number of mature live and dead trees (horizontal and vertical) at six different height classes (2–5 m, 6–10 m, 11–15 m, 16–20 m, 20–25 m, >25 m; Ehlers Smith et al., 2017a). Each point count of the 371 was sampled once during the wet (October 2021 to March 2021) and dry (April 2021 to September 2021) seasons. To determine the tree species richness and diversity, we identified all the trees within the plot to species level and identified the dominant and codominant plant species. The Gap Light Analysis Mobile Application (GLAMA) measured canopy openness, closure, and cover at each survey point. For every survey point, we used the mean height of all the vegetation classes (grass, herb, sapling or tree) to calculate the height heterogeneity index and used the mean percentage cover for the structural complexity using the Shannon–Weiner Diversity Index (SWDI) as follows: *H* = −∑ *p*_*i*_ ln (*p*_*i*_), where ‘*p*_*i*_’ is the proportion of the total foliage in the *i*th layer for a chosen horizontal layer (Bibby et al., 2000).

### 2.5 Data analyses

We quantified the bird's functional diversity using three indices: functional richness (Fric), functional evenness (FEve) and functional divergence (FDiv). Functional richness quantifies the total amount of functional space filled by a given community, which is the bird assemblage in this study (Villéger et al., 2008). Functional richness is independent of species' relative abundance and only increases if functionally different species are added to the community utilising more resources (Mason et al., 2005; Villéger et al., 2008). Functional evenness and divergence quantify how species occupy the functional space and account for their relative abundance, FEve measures the distance between species, and FDiv measures the species to the centre of the multidimensional trait space. Similarly, we created bird species matrices for every surveyed season (breeding and nonbreeding) using the abundance, presence and absence data. For each forest patch, we calculated FRic, FEve and FDiv for the whole bird community, forest specialists and forest generalists based on Gower functional dissimilarity using the function ‘dbFD’ in the package ‘FD’ (Laliberté et al., 2014). Using the z‐score formula, we standardised all the response variables to be comparable. Prior to analyses, we tested for spatial autocorrelation (Moran's *I*) using the package ‘ape’ in R (Dormann et al., 2007). Generalised linear models were performed to explore the influence of the retained explanatory variables on our response variable bird species richness (total number of species within the patch) Fric, FEve and FDiv (Table S2). Using the package ‘performance’ in R, we detected overdispersion for the count data model and used a negative binomial error distribution for all the models (Lüdecke et al., 2021). Using the Akaike Information Criterion (AIC), we defined the best model with ≤2 ΔAIC (Burnham & Anderson, 2004). We calculated McFadden's Pseudo *R* ^2^ for all the top models. To determine the effect of land use type (e.g., grassland, exotic tree plantation) adjacent to the forest patches and patch size on bird species composition across the 14 surveyed patches, we used a nonmetric dimensional scaling (NMDS) based on bird species abundance data. Bird species occurring in only one forest patch (outliers) were removed (*n* = 11); therefore, only 85 bird species were used for the NMDS. We analysed the data using the package ‘vegan’ and function ‘metaMDS’, Bray–Curtis distance matrix with two dimensions (Oksanen et al., 2022). To visualise the results, we used ggplot2. To test the difference in bird species composition in different patch sizes adjacent to different land use types, we used an analysis of similarity (ANOMIS) vegan package, Bray–Curtis and 9999 permutations. Lastly, we used the indicator species analysis in the package ‘indicspecies’ to test for bird species that are indicators of different forest patch sizes. We report bird species <0.05.

To assess the effects of habitat structural and compositional characteristics on the presence of the three selected forest specialists, we performed generalised linear models assuming a binomial error distribution using the packages ‘MASS’. Collinearity was checked and tested between all the explanatory variables using the variance inflation factors (VIF) using the package ‘car’. After excluding variables with higher VIF > 5 and correlation (*r* > .70) (i.e. canopy closure, canopy cover), eight variables were retained: percentage bare ground, leaf litter, grass, herbaceous, saplings, tree species richness, dead tree density and canopy openness. The structural characteristics were the explanatory variable, and the forest bird species' presence or absence (binary) data were used as a response variable. We used the Akaike Information for interpretation and selected the top models with ΔAIC ≤2 as the top models (Burnham & Anderson, 2004). To reduce uncertainty, we modelled averaged the parameters for all the models ΔAIC ≤2 as they are considered to be equal (Johnson & Omland, 2004).

## 3 RESULTS

### 3.1 Tree species composition and forest structure

We identified a total of 68 tree species belonging to 35 families and 56 genera in the KwaZulu‐Natal Midlands Mistbelt Forest patches (Table S3). Three of these tree species are presently listed as vulnerable, two as endangered and five are regarded as declining. The most dominant tree families were Flacourtiaceae (17%), Rutaceae (14%) and Celastraceae (11%). The total number of tree species varied between 60 (Karkloof) and 25 (Balgowan) across the studied regions, and the smallest patch (Waterfall Forest) had the least number of species (*n* = 5) (Table S4). Of the 68 tree species, only one species *Solanum mauritianum* was an alien invasive plant invading 11 forest patches except for Ingelabantwana Nature Reserve in Bulwer and Waterfall and Wakefield Forests in the Dargle (Table S4). The common forest tree species across sites were *Podocarpus/Afrocarpus* spp., *Xymalos monospora*, and the dominant understory species were *Carissa bispinosa, Diospyros whyteana*, and *Gymnosporia harveyana*. A total of 12 tree species (18%) were only identified in the Karkloof area, and six tree species (9%) were only identified in the Bulwer area (Table S4). Some species were only found in specific sites or fewer patches, including *Peddiea africana*, which was only identified in Balgowan, *Pittosporum viridiflorum* only in Bulwer and Dargle, and *Ocotea bullata* in Karkloof Nature Reserve and Marutswa Nature Reserve (Table S4).

### 3.2 Bird species composition

We recorded a total of 12,949 individuals, and 96 bird species belonging to 39 families across the 14 indigenous forest patches of the Mistbelt Forest in the Midlands of KwaZulu‐Natal (Tables S1 and S5). Eight bird species are regionally listed as either vulnerable, endangered or near threatened, and seven are listed globally (Tables S1 and S5). The recorded bird species differed in habitat association, feeding guild, body mass, feeding and nesting strategy (Table S1). They were a total of 39 (41%) forest habitat specialists and 57 (59%) generalists (Table S1). The most common trophic guild were insectivores (51%), and nest or cup was the dominant nesting strategy (61%) (Table S1). Bird body mass varied, ranging between 8 g and 4000 g. In the breeding season, there was a total of 91 bird species, and the most dominant bird species across all the forest patches were southern boubou *Laniarius ferrugineus* (*n* = 657), sombre greenbul *Andropadus importunus* (*n* = 557) and bar‐throated apalis *Apalis thoracica* (*n* = 487) (Table S5). In the nonbreeding season, there was a total of 68 bird species, and the most common species were the southern boubou (*n* = 656), southern double‐collared sunbird *Cinnyris chalybeus* (*n* = 538) and dark‐capped bulbul *Pycnonotus tricolor* (*n* = 475) (Table S5).

Some bird species were only found in specific sites or patches. For example, the eastern bronze‐naped pigeons *Columba delegorguei* were only in Karkloof Nature Reserve, and the crested guineafowl *Guttera edouardi* only in Karkloof forest patches. The number of bird species per forest patch were: Karkloof Nature Reserve (*n* = 67), Mbona Private Nature Reserve (*n* = 63), L'Abri (*n* = 45), Benvie Farm (*n* = 49), Rameron (*n* = 44), Milestone Forest Walk (*n* = 31), Maritzdaal (*n* = 64), Sharedown Forest (*n* = 45), Wakefield Forest (*n* = 20), Waterfall Forest (*n* = 16), Ingelabantwana Nature Reserve (*n* = 52), Xotsheyake Nature Reserve (*n* = 43), Marutswa Nature Reserve (*n* = 45), Nxumeni Forest (*n* = 53).

**FIGURE 2:**
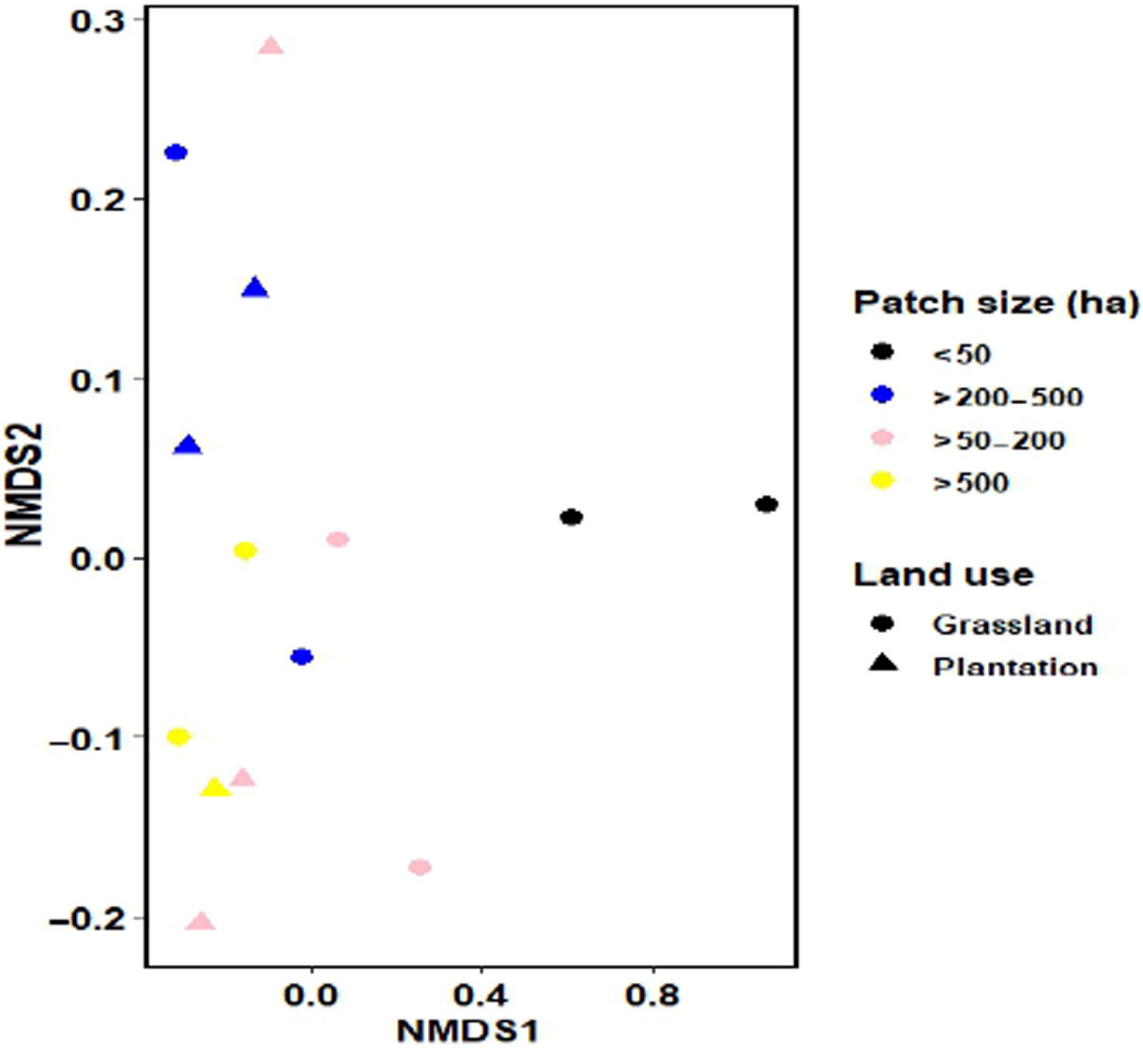
Nonmetric dimensional scaling (NMDS) of the bird species composition across 14 patches in four sites in the Midlands Mistbelt forests in South Africa (stress = 0.06; *k* = 2).

In the NMDS ordination based on the dissimilarity of the bird species composition around forest patches surrounded by commercial plantations and grassland matrix was not significantly different (*p* = .74; Figure 2). The bird species composition significantly differed in forest patches of different sizes (*p* < .05; Figure 2). We identified 40 bird species that are indicators for forest patches over 500 ha (Table S6). These included 22 forest bird specialists and 20 generalist bird species. Among the top forest specialist bird species strongly associated with forest patches >500 ha is the blue‐mantle crested flycatcher (*Trochocercus cyanomela*), Knysna turaco (*Tauraco corythaix*) and grey cuckooshrike (*Coracina caesia*) (Table S6). Forest generalists that are top indicators for forest patches >500 ha included the dark‐capped bulbul, tawny flanked prinia (*Prinia subflava*) and terrestrial brownbul (*Phyllastrephus terrestris*). Only one bird, the southern ground‐hornbill (*Bucorvus leadbeateri*), was identified as an indicator for patches that are 200–500 ha (Table S6).

### 3.3 Bird functional diversity and richness

**FIGURE 3:**
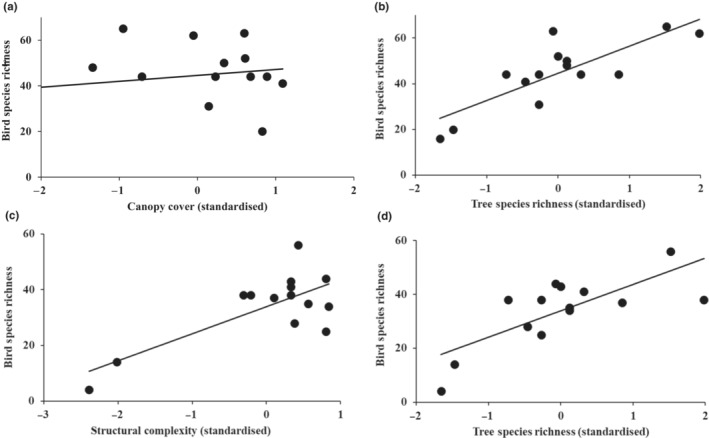
Relationship between bird species richness with (a) canopy cover, (b) tree species richness during the breeding season and (c) structural complexity and (d) c tree species richness during the non‐breeding season in selected Midlands Mistbelt Forest patches (*n* = 14) in KwaZulu‐Natal, South Africa.

The importance of the forest structure and composition characteristics influencing bird species diversity and richness differed across the surveyed seasons (Table S2). In both the breeding and nonbreeding season, tree species richness positively influenced bird species richness (Table 2, Figure 3). Forest structural complexity had a significant positive influence (*p* < .001) on the bird species richness in the nonbreeding season, and canopy cover had a nonsignificant positive influence in the breeding season (Figure 3). In the breeding season, FRic and FDiv for the whole community had a positive association with tree species richness, while FEve had a negative association with tree species richness (Table 3, Table S7). Functional divergence had an inconsistent response to tree species richness with seasonal differences.

**TABLE 2:** Generalised Linear Models (GLMs) averaged top models output based on ΔAIC rankings of the effects of tree species richness (TRic), structural complexity (SC), canopy cover (CC) and leaf litter (LL) on bird species richness and functional diversity (Fric, FEVe, FDiv) of avian communities of the Midlands Mistbelt forest patches in the breeding and non‐breeding season in KwaZulu‐Natal, South Africa.

	Response variable	Model structure	df	AIC	ΔAIC	Log‐likelihood	*R* ^2^
*Non‐breeding*							
Bird species richness	All birds	**TRic + SC (+)**	11	107.43	0.00	−99.43	.34
FRic	All birds	SC (+)	12	61.75	0.00	−55.75	.36
	Forest specialists	**SC (+)**	12	55.48	0.00	−49.48	.37
	Forest generalists	SC (+) + CC(−)	11	55.77	0.00	−47.77	.26
FEve	All birds	SC (−)	12	23.94	0.00	−17.94	.21
	Forest specialists	**SC (+)**	12	21.05	0.00	−16.05	.28
	Forest generalists	**CC (−)**	12	22.18	0.00	−17.18	.31
FDiv	All birds	TRic (−)	12	31.98	0.00	−25.978	.37
	Forest specialists	**SC (+)**	12	31.64	0.00	−25.64	.22
	Forest generalists	**CC (−)**	12	29.17	0.00	−23.15	.23
		SC (+)	12	29.17	0.00	−23.15	.23
*Breeding*							
Bird species richness	All birds	**TRic (+)**	12	109.20	0.00	−103.20	.37
FRic	All birds	TRic (+)	12	60.60	0.00	−54.60	.21
	Forest specialists	TRic (+)	12	56.94	0.00	−50.94	.35
	Forest generalists	TRic (+)	12	55.00	0.00	−49.00	.33
FEve	All birds	TRic (+)	12	25.33	0.00	−19.33	.21
	Forest generalists	**TRic (−)**	12	25.18	0.00	−19.15	.25
	Forest specialists	**TRic (+)**	12	23.43	0.00	−17.43	.34
FDiv	All birds	TRic (+)	12	31.91	0.00	−25.92	.17
		CC (−)	12	31.91	0.00	−25.92	.17
		LL (−)	12	31.91	0.00	−25.92	.17
		SC (−)	12	31.91	0.00	−25.91	.17
	Forest specialists	**TRIC (+)**	12	32.58	0.00	−26.57	.31
		**SC (+)**	12	32.58	0.00	−26.57	.31
	Forest generalists	**SC (+)**	12	30.24	0.00	−24.24	.24
		TRIC (+)	12	30.24	0.00	−24.24	.24

*Note*: This table only represents (ΔAIC = 0) though all top models (ΔAIC < 2) were averaged and considered further and *R* ^2^ indicates the top model performance (McFadden's Pseudo *R* ^2^).

**TABLE 3:** Generalised Linear Models (GLM) top model output of the effects of forest structure and composition characteristics influencing the occurrence of three forest specialists in the Midlands Mistbelt patches in KwaZulu‐Natal, South Africa.

Species	Variables	*p*‐Value	AICc	ΔAIC	*ω*_*i*_
Cape parrot *Poicephalus robustus*			306.65	0.00	0.42
	Canopy openness (+)	**5.32 e‐06**			
	Grass cover (−)	**.000**			
	Tree species richness (−)	**.001**			
	Snags (+)	.07			
	Sapling cover (+)	**.001**			
	Canopy openness (+)	**2e‐16**	307.48	0.83	0.39
	Tree species richness (−)	**.001**			
	Saplings (+)	**.001**			
	Herbaceous cover (+)	.22			
Forest canary *Crithagra scotops*	Tree species richness (+)	**2e‐16**	302.95	0.00	0.37
	Herbaceous cover (+)	**2.01e‐06**			
Orange ground‐thrush *Geokichla gurneyi*			96.29	0.00	0.33
	Herbaceous cover (−)	**.001**			
	Tree species richness (+)	**3.32e‐06**			
	Bareground (+)	.45	97.55	1.26	0.16
	Tree species richness (+)	3.66e‐06			
	Herbaceous cover (−)	.04			

*Note*: Bold indicates significance. Significant codes: <0 ‘***’ .001 ‘**’ .01 ‘*’ .05.

Abbreviations: AICc, Akaike's Information Criterion; ΔAIC, Delta AIC; *ω*_*i*_, Akaike weights.

During the breeding and nonbreeding seasons, forest bird specialists' functional diversity was, respectively, influenced by structural complexity and tree species richness (Table 2, Table S7). Forest generalists' diversity was mainly influenced by forest canopy cover and structural complexity (Table 2, Table S7). During the breeding season, forest generalist bird species FDiv and FEve had a positive association with structural complexity (Table 2). During the nonbreeding season, canopy cover had a significant (*p* < .05) negative influence on forest generalist birds FEve (Table 2). Structural complexity was an important variable for forest specialist bird species in both seasons (Table 2). In contrast, leaf litter was important during the breeding season and water cover during the non‐breeding season (Table 2, Table S7). During the breeding season, the whole bird community, forest generalists and forest specialists FDiv had a negative association with leaf litter (Table S7). During the nonbreeding season whole bird community and forest bird specialist FDiv had a negative association with water cover, while forest generalists had a positive association (Table S7).

### 3.4 Habitat use

Individual forest‐dependent bird species varied in their response to forest structural characteristics and compositional characteristics (Table 3). The Cape parrots were only present in the Bulwer area in all four patches and were more abundant in Ingelabantwana Nature Reserve and Marutswa Nature Reserve. The percentage cover of saplings had a significant positive influence on the presence of Cape parrots, and the grass cover had a significant negative effect (Table 3). Also, tree species richness negatively influenced the presence of the Cape parrot, while canopy openness had a positive effect (Table 3). Forest patches occupied by Cape parrots were compositionally similar. The dominant tree species in these patches were: *Podocarpus/Afrocarpus* spp., *Ptaeroxylon obliquum*, and the dominant understory were *Gymnosporia harveyana*, and *Diospyros whyteana*. The herbaceous cover and tree species richness influenced the forest canary and the orange ground‐thrush presence. The forest canary was present in forest patches with different tree species compositions (Nxumeni, Karkloof Nature Reserve, Mbona Private Nature Reserve and Maritzdaal).

## 4 DISCUSSION

As the transformation of habitats increases, changing the structure and composition of forest habitats, it is important to understand the influence on the avian community. In this study, we evaluated the responses of bird species richness, three functional diversity indices and selected forest specialists to vegetation structure and composition characteristics. We demonstrated the importance of forest structure and composition in shaping the bird community structure, richness, functional diversity and forest specialists' species‐specific responses. Our results showed the importance of tree species richness in bird species richness and diversity. Also, we showed that species‐specific responses to local scale structural and compositional attributes do not scale up to the whole community response. This supported our hypothesis that forest specialist bird species richness responds differently to vegetation structure and composition characteristics.

### 4.1 Bird species richness and forest specialists' responses to forest structure and vegetation

Local tree species richness was a significant factor that positively affected bird species richness in the Mistbelt Forest patches of KwaZulu‐Natal. This positive response to high tree diversity by bird species has been reported in previous studies for bird species (Gil‐Tena et al., 2007; Regnery et al., 2013). Tree species richness influence on species has been shown for other taxa, including amphibians (McKenny et al., 2006; Sankararaman et al., 2021) and mammals (Regnery et al., 2013; Wells et al., 2007). Tree species richness contributes to the heterogeneity and spatial complexity of the forests by providing more microhabitats and resources. For example, increased tree species richness provides more nesting and food resources (Gil‐Tena et al., 2007; Ong'ondo et al., 2022). For insectivorous species like the orange ground‐thrush *G. gurneyi*, high tree species richness increases insect abundance over different seasons (Bereczki et al., 2014). The orange ground‐thrush is a generalist insectivore feeding mainly on insects and earthworms (Hockey et al., 2005); therefore, different trees provide them with different insects as different tree microhabitats are associated with different insect communities. While it appears that forest tree species richness positively influenced the presence of forest specialist birds, the Cape parrot presence had a negative association with tree species‐rich forest patches. Past studies show that the richness of tree species does not influence bird species that depend solely on a specific tree species microhabitat. Cape parrots depend primarily on *Podocarpus/ Afrocarpus* spp. for nesting and feeding (Leaver et al., 2022; Wirminghaus et al., 1999, 2001, 2002). This was corroborated in the present study by the absence of Cape parrots in forest patches with high tree diversity. Exotic pecan nut *Carya illinoinensis* plantations outside forests provide Cape parrots with foraging resources during autumn (Symes & Downs, 2002; Wimberger et al., 2023). It has been argued that forest bird specialists prefer natural forests more than surrounding landscapes for supplementary resources (Ong'ondo et al., 2022; Porro et al., 2020). The present study did not consider the landscape attributes, which may be more important in habitat use by Cape parrots. Assessing the landscape context will give more insights into the factors influencing the Cape parrot's habitat use and selection. Intense logging history in the KwaZulu‐Natal Midlands Mistbelt forests decreased the dominance of *Podocarpus/Afrocarpus* spp. (Adie et al., 2013). This can explain the Cape parrot's absence in unoccupied patches in the present study. Although we did not quantify the logging intensity, Karkloof forest patches were heavily logged (J Geekie pers. comm., unpublished data). While surrounding communities used some Bulwer forest patches, they were not exploited by the early settlers because these patches were difficult to access (King, 1941). *Podocarpus/ Afrocarpus* spp. cannot recover quickly, especially in heavily logged forest patches, and the remaining tree species are limited by seed dispersal distances (Wirminghaus et al., 1999). Downs et al. (2014) attributed the Cape parrot decline in the Karkloof area (Benvie and Mbona Private Nature Reserve) to food and nest site shortages. Structural complexity and canopy cover were the main forest attributes influencing bird species richness. Structural complexity has been previously linked to microhabitat formation and ecological niche availability (Castaño‐Villa et al., 2014; Ehlers Smith et al., 2018). Structural heterogeneity has been related to increased available microhabitats for foraging (Şekercioḡlu et al., 2002) and nesting sites (Martin et al., 2004). Structural characteristics influencing habitat utilisation by forest specialists were different for different species. These results are consistent with previous studies showing that different structural characteristics influence forest species occupancy as species have different microhabitat requirements (Braunisch et al., 2019; Gumede, Ehlers Smith, Ngcobo, et al., 2022). In our study, Cape parrots used forest patches with a dense shrubby understory, a high number of snags, and low grass cover. Our findings reinforce the positive relationship of the Cape parrot with dead trees/snags, as previously reported (Downs & Symes, 2004). Our data suggested a strong negative influence of the herbaceous cover on the orange ground‐thrush. Gumede, Ehlers Smith, Ngcobo, et al. (2022) showed that this species prefers an open understory with a high leaf litter cover. In our study, the herbaceous cover positively influenced the forest canary, consistent with the dietary as a granivorous bird (Hockey et al., 2005) and nesting requirements for dense leafy foliage (Tarboton, 2001). The response of forest‐specialised bird species to tree diversity and forest structural characteristics cannot be generalised from functional group responses. Therefore, conservation‐oriented approaches to forest management must assess species‐specific and community responses (Basile et al., 2021).

### 4.2 Functional diversity responses to vegetation diversity and forest structural attributes

Our results showed that bird species, local vegetation structure and diversity influence bird functional diversity. In the present study, functional richness was higher in forest patches with higher tree species richness for forest generalists and specialist birds during the breeding season. This positive response of functional richness to tree diversity is attributed to increased resource availability and stand structure complexity from different tree species (Díaz, 2006; Ehbrecht et al., 2017). During the nonbreeding season, functional richness had a positive association with structural complexity for the whole bird community, while functional evenness and divergence had a negative association. A similar relationship was observed in Australia, where these bird functional diversity indices showed opposing responses to vegetation structure and tree species richness (Sitters et al., 2016). During the nonbreeding season, forest structural complexity positively affected the diversity of forest bird specialists (functional eveness, divergence) and generalists (divergence) in the present study. Increased functional evenness reflects the efficiency of resource partitioning (Hillebrand et al., 2008) and increased ecosystem functioning in structurally complex forest patches (Mason et al., 2005). These results emphasise the importance of structurally complex forest patches to allow more niches to be occupied by bird species and resource heterogeneity. Therefore, site‐scale habitat destruction of forest systems will affect species diversity and the functionality of forests (Bogoni et al., 2020). Focussing on the total number of bird species without considering bird functional diversity or specific indices can lead to misleading forest management strategies because of the different responses of the diversity indices (Lelli et al., 2019; Matuoka et al., 2020).

## 5 CONCLUSIONS

Site scale structural and vegetation composition characteristics are important in maintaining high species richness and functional richness. Our study highlighted important local scale characteristics that can be managed to maintain bird species richness and forest specialists' occurrence. Tree species richness played an important role in maintaining high bird species richness, diversity and habitat use by forest specialists. Preserving local scale structural characteristics and promoting high tree species richness in the Midlands Mistbelt Forest will help maintain high species richness and diversity. This study indicated that forest‐dependent bird species have distinct responses to vegetation composition and forest structural attributes. This indicates that management approaches that target community responses may not meet the requirement of vulnerable forest specialists. The importance of vegetation diversity and forest structural attributes varied between the forest bird species and diversity indices. Consideration of community and species‐specific responses may improve conservation and biodiversity management. Our results suggest that the Bulwer Forest complex patches especially are important for the persistence of the remnant subpopulation of the endangered Cape parrot in the Midlands Mistbelt Forests of KwaZulu‐Natal. Although these forest patches are protected and managed by Ezemvelo KwaZulu‐Natal Wildlife (the government conservation parastatal) and the Department of Environment, Forest and Fisheries, they continue to be used by surrounding communities in the landscape mosaic, mainly for animal grazing, and these activities are not monitored (NB pers. obs.). It is important to implement sustainable use initiatives to ensure the conservation of Cape parrots in these forest patches. Our study confirmed that tree species richness should be maintained and is important in the conservation of forest specialists, bird species richness and functional richness. Assessing scale structural and vegetation characteristics is adequate for the management of forest patches at a local scale, especially if they are privately owned (Gil‐Tena et al., 2007). However, we still consider the need to explore the landscape characteristics further, especially to understand habitat selection by Cape parrots.

## AUTHOR CONTRIBUTIONS

**Nasiphi Bitani:** Conceptualization (equal); data curation (equal); formal analysis (lead); investigation (lead); methodology (lead); project administration (equal); validation (lead); visualization (lead); writing – original draft (lead); writing – review and editing (equal). **Craig Cordier:** Conceptualization (equal); data curation (equal); investigation (equal); methodology (equal); validation (equal); writing – review and editing (equal). **David A. Ehlers Smith:** Conceptualization (equal); formal analysis (equal); supervision (equal); writing – review and editing (equal). **Yvette C. Ehlers Smith:** Conceptualization (equal); supervision (equal); writing – review and editing (equal). **Colleen T. Downs:** Conceptualization (equal); data curation (equal); funding acquisition (lead); investigation (equal); methodology (equal); project administration (lead); resources (lead); supervision (lead); writing – review and editing (equal).

## CONFLICT OF INTEREST STATEMENT

We declare no conflict of interest in connection with the work submitted.

## Supporting information


Table S1

## Data Availability

Data for this study belong to the University of KwaZulu‐Natal. Data are presented in the Supplementary Information and archived in Dryad (DOI: https://doi.org/10.5061/dryad.f1vhhmh2s).
