# Supplementary material for: Avian species functional diversity and habitat use: The role of forest structural attributes and tree diversity in the Midlands Mistbelt Forests of KwaZulu‐Natal, South Africa
Source: Ecol Evol. 2023 Aug 31;13(9):e10439. doi: 10.1002/ece3.10439 (PMC10469004; doi:10.1002/ece3.10439)
Supplement: Supplementary file 1 — Table S1 [file ECE3-13-e10439-s001.docx]

**Avian species functional diversity and habitat use: the role of forest structural attributes and tree diversity in the Midlands Mistbelt forests of KwaZulu-Natal, South Africa**

Nasiphi Bitani^1^, Craig Cordier^1^, David A. Ehlers Smith^1^, Yvette C. Ehlers Smith^1, 2^, Colleen T. Downs^1^*

**SUPPLEMENTARY INFORMATION**

**Supplementary Information Table S1** Functional traits of the bird species by Hockey et al. (2005) in the present study.

|  | **Dietary guild** | | | | | | | **Foraging strategy** | | | | | | | **Nesting strategy** | | | | **Habitat specificity** | |
| --- | --- | --- | --- | --- | --- | --- | --- | --- | --- | --- | --- | --- | --- | --- | --- | --- | --- | --- | --- | --- |
| **Species name** | Body mass | Carnivory | Frugivory | Omnivory | Nectarivory | Granivory | Insectivory | Harvest | Terrestrial probe | Arboreal probe | Glean | Hawk | Perch and swoop | Various | Ground | Ball or cup | Cavity | Platform | Generalist | Specialists |
| *Andropadus importunus* | 31 | 0 | 1 | 0 | 0 | 0 | 0 | 1 | 0 | 0 | 0 | 0 | 0 | 0 | 0 | 1 | 0 | 0 | 1 | 0 |
| *Apalis flavida* | 8 | 0 | 0 | 0 | 0 | 0 | 1 | 0 | 0 | 0 | 1 | 0 | 0 | 0 | 0 | 1 | 0 | 0 | 1 | 0 |
| *Apalis thoracica* | 10.5 | 0 | 0 | 0 | 0 | 0 | 1 | 0 | 0 | 0 | 1 | 0 | 0 | 0 | 0 | 1 | 0 | 0 | 0 | 1 |
| *Apaloderma narina* | 67 | 0 | 0 | 0 | 0 | 0 | 1 | 0 | 0 | 0 | 0 | 0 | 1 | 0 | 0 | 0 | 1 | 0 | 0 | 1 |
| *Aplopelia larvata* | 150 | 0 | 0 | 0 | 0 | 1 | 0 | 0 | 1 | 0 | 0 | 0 | 0 | 0 | 0 | 0 | 0 | 1 | 0 | 1 |
| *Batis capensis* | 12 | 0 | 0 | 0 | 0 | 0 | 1 | 0 | 0 | 0 | 1 | 0 | 0 | 0 | 0 | 1 | 0 | 0 | 0 | 1 |
| *Bostrychia hagedash* | 1250 | 0 | 0 | 0 | 0 | 0 | 1 | 0 | 1 | 0 | 0 | 0 | 0 | 0 | 0 | 1 | 0 | 0 | 1 | 0 |
| *Bradypterus barratti* | 15 | 0 | 0 | 0 | 0 | 0 | 1 | 0 | 0 | 0 | 0 | 0 | 0 | 0 | 1 | 1 | 0 | 0 | 1 | 0 |
| *Bucorvus leadbeateri* | 3770 | 0 | 0 | 1 | 0 | 0 | 0 | 0 | 0 | 0 | 0 | 0 | 0 | 1 | 1 | 0 | 0 | 0 | 1 | 0 |
| *Buteo trizonatus* | 660 | 1 | 0 | 0 | 0 | 0 | 0 | 0 | 0 | 0 | 0 | 0 | 1 | 0 | 0 | 1 | 0 | 0 | 0 | 1 |
| *Bycanistes bunicator* | 644 | 0 | 1 | 0 | 0 | 0 | 0 | 1 | 0 | 0 | 0 | 0 | 0 | 0 | 0 | 0 | 1 | 0 | 0 | 1 |
| *Camaroptera brachyura* | 11 | 0 | 0 | 0 | 0 | 0 | 1 | 0 | 0 | 0 | 1 | 0 | 0 | 0 | 0 | 1 | 0 | 0 | 0 | 1 |
| *Campephaga flava* | 32 | 0 | 0 | 0 | 0 | 0 | 1 | 0 | 0 | 0 | 1 | 0 | 0 | 0 | 0 | 1 | 0 | 0 | 1 | 0 |
| *Campethera abingoni* | 70 | 0 | 0 | 0 | 0 | 0 | 1 | 0 | 0 | 1 | 0 | 0 | 0 | 0 | 0 | 0 | 1 | 0 | 0 | 1 |
| *Campethera notata* | 62 | 0 | 0 | 0 | 0 | 0 | 1 | 0 | 0 | 1 | 0 | 0 | 0 | 0 | 0 | 0 | 1 | 0 | 0 | 1 |
| *Centropus burchelliii* | 180 | 1 | 0 | 0 | 0 | 0 | 0 | 0 | 1 | 0 | 0 | 0 | 0 | 0 | 0 | 1 | 0 | 0 | 1 | 0 |
| *Cercotrichas leucophrys* | 17.9 | 0 | 0 | 0 | 0 | 0 | 1 | 0 | 1 | 0 | 0 | 0 | 0 | 0 | 0 | 1 | 0 | 0 | 1 | 0 |
| *Chalcomitra amethystina* | 15 | 0 | 0 | 0 | 1 | 0 | 0 | 1 | 0 | 0 | 0 | 0 | 0 | 0 | 0 | 1 | 0 | 0 | 1 | 0 |
| *Chrysococcyx capreus* | 35 | 0 | 0 | 0 | 0 | 0 | 1 | 0 | 0 | 0 | 0 | 0 | 1 | 0 | 0 | 1 | 0 | 0 | 0 | 1 |
| *Chrysococcyx caprius* | 30 | 0 | 0 | 0 | 0 | 0 | 1 | 0 | 0 | 0 | 1 | 0 | 0 | 0 | 0 | 1 | 0 | 0 | 1 | 0 |
| *Chrysococcyx klaas* | 26 | 0 | 0 | 0 | 0 | 0 | 1 | 0 | 0 | 0 | 1 | 0 | 0 | 0 | 0 | 1 | 0 | 0 | 1 | 0 |
| *Cinnyris chalybeus* | 8 | 0 | 0 | 0 | 1 | 0 | 0 | 0 | 0 | 0 | 0 | 0 | 0 | 1 | 0 | 1 | 0 | 0 | 1 | 0 |
| *Cisticola aberrans* | 13 | 0 | 0 | 0 | 0 | 0 | 1 | 0 | 0 | 0 | 1 | 0 | 0 | 0 | 0 | 1 | 0 | 0 | 1 | 0 |
| *Clamatar jacobinus* | 81 | 0 | 0 | 0 | 0 | 0 | 1 | 0 | 0 | 0 | 1 | 0 | 0 | 0 | 0 | 1 | 0 | 0 | 1 | 0 |
| *Coccopygia melanotis* | 6.5 | 0 | 0 | 0 | 0 | 1 | 0 | 1 | 0 | 0 | 0 | 0 | 0 | 0 | 0 | 1 | 0 | 0 | 1 | 0 |
| *Colius striatus* | 55 | 0 | 1 | 0 | 0 | 0 | 0 | 1 | 0 | 0 | 0 | 0 | 0 | 0 | 0 | 1 | 0 | 0 | 1 | 0 |
| *Columba arquatrix* | 407 | 0 | 1 | 0 | 0 | 0 | 0 | 1 | 0 | 0 | 0 | 0 | 0 | 0 | 0 | 0 | 0 | 1 | 0 | 1 |
| *Columba delegorguei* | 160 | 0 | 1 | 0 | 0 | 0 | 0 | 1 | 0 | 0 | 0 | 0 | 0 | 0 | 0 | 0 | 0 | 1 | 0 | 1 |
| *Coracina caesia* | 60 | 0 | 0 | 0 | 0 | 0 | 1 | 0 | 0 | 0 | 1 | 0 | 0 | 0 | 0 | 1 | 0 | 0 | 0 | 1 |
| *Cossypha caffra* | 28 | 0 | 0 | 0 | 0 | 0 | 1 | 0 | 1 | 0 | 0 | 0 | 0 | 0 | 0 | 1 | 0 | 0 | 1 | 0 |
| *Cossypha dichroa* | 46 | 0 | 0 | 0 | 0 | 0 | 1 | 0 | 1 | 0 | 0 | 0 | 0 | 0 | 0 | 0 | 0 | 0 | 0 | 1 |
| *Cossypha natalensis* | 32 | 0 | 0 | 0 | 0 | 0 | 1 | 0 | 1 | 0 | 0 | 0 | 0 | 0 | 0 | 1 | 0 | 0 | 1 | 0 |
| *Crithagra scotops* | 15 | 0 | 0 | 0 | 0 | 1 | 0 | 1 | 0 | 0 | 0 | 0 | 0 | 0 | 0 | 1 | 0 | 0 | 0 | 1 |
| *Crithagra sulphurata* | 17 | 0 | 0 | 1 | 0 | 0 | 0 | 0 | 0 | 0 | 0 | 0 | 0 | 1 | 0 | 0 | 0 | 1 | 1 | 0 |
| *Cuculus clamosus* | 90 | 0 | 0 | 0 | 0 | 0 | 1 | 0 | 0 | 0 | 0 | 1 | 0 | 0 | 0 | 1 | 0 | 0 | 1 | 0 |
| *Cuculus solitarius* | 75 | 0 | 0 | 0 | 0 | 0 | 1 | 0 | 0 | 0 | 1 | 0 | 0 | 0 | 0 | 1 | 0 | 0 | 0 | 1 |
| *Cyanomitra olivacea* | 11.5 | 0 | 0 | 0 | 1 | 0 | 0 | 1 | 0 | 0 | 0 | 0 | 0 | 0 | 0 | 1 | 0 | 0 | 0 | 1 |
| *Dendropicos griseocephalus* | 45 | 0 | 0 | 0 | 0 | 0 | 1 | 0 | 0 | 1 | 0 | 0 | 0 | 0 | 0 | 0 | 1 | 0 | 0 | 1 |
| *Dicrurus adsimilis* | 43.75 | 0 | 0 | 0 | 0 | 0 | 1 | 0 | 0 | 0 | 0 | 1 | 0 | 0 | 0 | 1 | 0 | 0 | 1 | 0 |
| *Dryoscopus cubla* | 26 | 0 | 0 | 0 | 0 | 0 | 1 | 0 | 0 | 0 | 1 | 0 | 0 | 0 | 0 | 1 | 0 | 0 | 1 | 0 |
| *Estrilda astrild* | 8 | 0 | 0 | 0 | 0 | 1 | 0 | 1 | 0 | 0 | 0 | 0 | 0 | 0 | 0 | 1 | 0 | 0 | 1 | 0 |
| *Guttera edouardii* | 1300 | 0 | 0 | 1 | 0 | 0 | 0 | 0 | 0 | 0 | 0 | 0 | 0 | 1 | 1 | 0 | 0 | 0 | 0 | 1 |
| *Hedysdipna collaris* | 8 | 0 | 0 | 0 | 1 | 0 | 0 | 1 | 0 | 0 | 0 | 0 | 0 | 0 | 0 | 1 | 0 | 0 | 0 | 1 |
| *Indicator minor* | 28 | 0 | 0 | 0 | 0 | 1 | 0 | 0 | 0 | 0 | 1 | 0 | 0 | 0 | 0 | 0 | 1 | 0 | 1 | 0 |
| *Indicator variegatus* | 48 | 0 | 0 | 0 | 0 | 0 | 1 | 0 | 0 | 0 | 0 | 1 | 0 | 0 | 0 | 0 | 1 | 0 | 0 | 1 |
| *Ispidina picta* | 15 | 0 | 0 | 0 | 0 | 0 | 1 | 0 | 0 | 0 | 0 | 0 | 1 | 0 | 1 | 0 | 0 | 0 | 0 | 1 |
| *Lagonostricta rhodopareia* | 10.3 | 0 | 0 | 0 | 0 | 1 | 0 | 1 | 0 | 0 | 0 | 0 | 0 | 0 | 0 | 1 | 0 | 0 | 1 | 0 |
| *Laniarius ferrugineus* | 60 | 0 | 0 | 0 | 0 | 0 | 1 | 0 | 0 | 0 | 0 | 1 | 0 | 0 | 0 | 1 | 0 | 0 | 1 | 0 |
| *Lanius collaris* | 40 | 0 | 0 | 1 | 0 | 0 | 0 | 0 | 0 | 0 | 0 | 0 | 0 | 1 | 0 | 1 | 0 | 0 | 0 | 1 |
| *Lioptilus nigricapillus* | 31 | 0 | 1 | 0 | 0 | 0 | 0 | 1 | 0 | 0 | 0 | 0 | 0 | 0 | 0 | 1 | 0 | 0 | 0 | 1 |
| *Lophaetus occipitalis* | 1055 | 1 | 0 | 0 | 0 | 0 | 0 | 0 | 0 | 0 | 0 | 0 | 1 | 0 | 0 | 1 | 0 | 0 | 1 | 0 |
| *Lybius torquatus* | 54 | 0 | 1 | 0 | 0 | 0 | 0 | 1 | 0 | 0 | 0 | 0 | 0 | 0 | 0 | 0 | 1 | 0 | 1 | 0 |
| *Malaconotus blanchoti* | 77 | 0 | 0 | 0 | 0 | 0 | 1 | 0 | 1 | 0 | 0 | 0 | 0 | 0 | 0 | 1 | 0 | 0 | 1 | 0 |
| *Melaenornis pammelaina* | 30 | 0 | 0 | 0 | 0 | 0 | 1 | 0 | 0 | 0 | 0 | 0 | 1 | 0 | 0 | 1 | 0 | 0 | 1 | 0 |
| *Muscicapa adusta* | 11 | 0 | 0 | 0 | 0 | 0 | 1 | 0 | 0 | 0 | 0 | 1 | 0 | 0 | 0 | 1 | 0 | 0 | 1 | 0 |
| *Muscicapa caerulescens* | 16.5 | 0 | 0 | 0 | 0 | 0 | 1 | 0 | 0 | 0 | 0 | 1 | 0 | 0 | 0 | 1 | 0 | 0 | 0 | 1 |
| *Myioparus plumbeus* | 21 | 0 | 0 | 0 | 0 | 0 | 1 | 0 | 0 | 0 | 1 | 0 | 0 | 0 | 0 | 1 | 0 | 0 | 1 | 0 |
| *Nectarinia fanosa* | 15 | 0 | 0 | 0 | 1 | 0 | 0 | 1 | 0 | 0 | 0 | 0 | 0 | 0 | 0 | 1 | 0 | 0 | 1 | 0 |
| *Numida meleagris* | 1350 | 0 | 0 | 1 | 0 | 0 | 0 | 0 | 0 | 0 | 0 | 0 | 0 | 1 | 1 | 0 | 0 | 0 | 1 | 0 |
| *Orious larvatus* | 65 | 0 | 0 | 0 | 1 | 0 | 0 | 0 | 0 | 0 | 0 | 0 | 0 | 1 | 0 | 1 | 0 | 0 | 1 | 0 |
| *Onychognathus morio* | 140 | 0 | 1 | 0 | 0 | 0 | 0 | 1 | 0 | 0 | 0 | 0 | 0 | 0 | 0 | 1 | 0 | 0 | 1 | 0 |
| *Parus niger* | 21 | 0 | 0 | 0 | 0 | 0 | 1 | 0 | 0 | 0 | 1 | 0 | 0 | 0 | 0 | 0 | 1 | 0 | 1 | 0 |
| *Phoeniculus purpureus* | 76 | 0 | 0 | 0 | 0 | 0 | 1 | 0 | 0 | 1 | 0 | 0 | 0 | 0 | 0 | 0 | 1 | 0 | 1 | 0 |
| *Phyllastrephus terrestris* | 31.5 | 0 | 0 | 0 | 0 | 0 | 1 | 0 | 0 | 0 | 0 | 0 | 0 | 0 | 0 | 1 | 0 | 0 | 1 | 0 |
| *Phylloscopus ruficapilla* | 8 | 0 | 0 | 0 | 0 | 0 | 1 | 0 | 0 | 0 | 1 | 0 | 0 | 0 | 1 | 0 | 0 | 0 | 0 | 1 |
| *Ploceous bicolor* | 35 | 0 | 0 | 0 | 0 | 0 | 1 | 0 | 0 | 0 | 1 | 0 | 0 | 0 | 0 | 1 | 0 | 0 | 0 | 1 |
| *Ploceous ocularis* | 30 | 0 | 0 | 1 | 0 | 0 | 0 | 0 | 0 | 0 | 0 | 0 | 0 | 1 | 0 | 1 | 0 | 0 | 1 | 0 |
| *Ploceus velatus* | 34 | 0 | 0 | 0 | 0 | 0 | 0 | 0 | 0 | 0 | 0 | 0 | 0 | 0 | 0 | 0 | 0 | 0 | 0 | 0 |
| *Pogoniulus pusillus* | 17 | 0 | 1 | 0 | 0 | 0 | 0 | 1 | 0 | 0 | 0 | 0 | 0 | 0 | 0 | 0 | 1 | 0 | 1 | 0 |
| *Pogonocichla stellata* | 21 | 0 | 0 | 0 | 0 | 0 | 1 | 0 | 0 | 0 | 1 | 0 | 0 | 0 | 0 | 1 | 0 | 0 | 0 | 1 |
| *Poicephalus robustus* | 300 | 0 | 1 | 0 | 0 | 0 | 0 | 1 | 0 | 0 | 0 | 0 | 0 | 0 | 0 | 0 | 1 | 0 | 0 | 1 |
| *Polemaetus bellicosus* | 4000 | 1 | 0 | 0 | 0 | 0 | 0 | 0 | 0 | 0 | 0 | 1 | 0 | 0 | 0 | 0 | 0 | 1 | 1 | 0 |
| *Prinia subflava* | 9.5 | 0 | 0 | 1 | 0 | 0 | 0 | 0 | 0 | 0 | 0 | 0 | 0 | 1 | 0 | 0 | 0 | 0 | 1 | 0 |
| *Pternistis natalensis* | 445 | 0 | 0 | 1 | 0 | 0 | 0 | 0 | 0 | 0 | 0 | 0 | 0 | 1 | 1 | 0 | 0 | 0 | 1 | 0 |
| *Pternitsis afer* | 640 | 0 | 0 | 1 | 0 | 0 | 0 | 0 | 0 | 0 | 0 | 0 | 0 | 1 | 1 | 0 | 0 | 0 | 0 | 1 |
| *Pternitsis swainsonii* | 585 | 0 | 0 | 1 | 0 | 0 | 0 | 0 | 0 | 0 | 0 | 0 | 0 | 1 | 1 | 0 | 0 | 0 | 1 | 0 |
| *Pycnonotus tricolor* | 37.3 | 0 | 1 | 0 | 0 | 0 | 0 | 1 | 0 | 0 | 0 | 0 | 0 | 0 | 0 | 1 | 0 | 0 | 1 | 0 |
| *Sarothruta elegans* | 45 | 0 | 0 | 0 | 0 | 0 | 1 | 0 | 1 | 0 | 0 | 0 | 0 | 0 | 1 | 0 | 0 | 0 | 0 | 1 |
| *Spermestes cucullata* | 10 | 0 | 0 | 0 | 0 | 0 | 1 | 0 | 0 | 0 | 0 | 0 | 0 | 1 | 0 | 1 | 0 | 0 | 1 | 0 |
| *Stephanoaetus coronatus* | 34000 | 1 | 0 | 0 | 0 | 0 | 0 | 0 | 0 | 0 | 0 | 0 | 1 | 0 | 0 | 0 | 0 | 1 | 0 | 1 |
| *Streptopelia capicola* | 150 | 0 | 0 | 1 | 0 | 0 | 0 | 0 | 0 | 0 | 0 | 0 | 0 | 1 | 0 | 0 | 0 | 1 | 1 | 0 |
| *Streptopelia semitorquata* | 235 | 0 | 0 | 0 | 0 | 1 | 0 | 0 | 1 | 0 | 0 | 0 | 0 | 0 | 0 | 0 | 0 | 1 | 1 | 0 |
| *Streptopelia senegalensis* | 100 | 0 | 0 | 0 | 0 | 1 | 0 | 0 | 1 | 0 | 0 | 0 | 0 | 0 | 0 | 0 | 0 | 0 | 1 | 0 |
| *Sylvietta rufescens* | 11 | 0 | 0 | 0 | 0 | 0 | 1 | 0 | 1 | 0 | 0 | 0 | 0 | 0 | 0 | 0 | 0 | 1 | 1 | 0 |
| *Tauraco corythaix* | 300 | 0 | 1 | 0 | 0 | 0 | 0 | 1 | 0 | 0 | 0 | 0 | 0 | 0 | 0 | 0 | 0 | 1 | 0 | 1 |
| *Telophorus olivaceus* | 33 | 0 | 0 | 0 | 0 | 0 | 1 | 0 | 0 | 0 | 1 | 0 | 0 | 0 | 0 | 1 | 0 | 0 | 0 | 1 |
| *Telophorus viridis* | 37 | 0 | 0 | 0 | 0 | 0 | 1 | 0 | 0 | 0 | 1 | 0 | 0 | 0 | 0 | 1 | 0 | 0 | 1 | 0 |
| *Terpsiphone viridis* | 14.25 | 0 | 0 | 0 | 0 | 0 | 1 | 0 | 0 | 0 | 0 | 1 | 0 | 0 | 0 | 1 | 0 | 0 | 1 | 0 |
| *Tockus alboterminatus* | 225 | 0 | 0 | 1 | 0 | 0 | 0 | 0 | 0 | 0 | 0 | 0 | 0 | 1 | 0 | 0 | 1 | 0 | 0 | 1 |
| *Treron calvus* | 235 | 0 | 1 | 0 | 0 | 0 | 0 | 1 | 0 | 0 | 0 | 0 | 0 | 0 | 0 | 0 | 0 | 1 | 1 | 0 |
| *Trochocercus cyanomelas* | 10 | 0 | 0 | 0 | 0 | 0 | 1 | 0 | 0 | 0 | 0 | 1 | 0 | 0 | 0 | 1 | 0 | 0 | 0 | 1 |
| *Turdus olivaceous* | 66 | 0 | 0 | 0 | 0 | 0 | 1 | 0 | 1 | 0 | 0 | 0 | 0 | 0 | 0 | 1 | 0 | 0 | 1 | 0 |
| *Turtur tympanistria* | 71 | 0 | 0 | 0 | 0 | 1 | 0 | 1 | 0 | 0 | 0 | 0 | 0 | 0 | 0 | 1 | 0 | 0 | 0 | 1 |
| *Upupa africana* | 53 | 0 | 0 | 0 | 0 | 0 | 1 | 0 | 1 | 0 | 0 | 0 | 0 | 0 | 0 | 0 | 1 | 0 | 1 | 0 |
| *Geokichla gurneyi* | 68 | 0 | 0 | 0 | 0 | 0 | 1 | 0 | 0 | 0 | 0 | 0 | 0 | 0 | 1 | 0 | 0 | 0 | 0 | 1 |
| *Zosterops virens* | 13.5 | 0 | 0 | 1 | 0 | 0 | 0 | 0 | 0 | 0 | 0 | 0 | 0 | 1 | 0 | 1 | 0 | 0 | 1 | 0 |

**Supplementary Information Table S2** Generalised Linear Models (GLMs) top models output of the effects of tree species richness (TRic), structural complexity (SC), canopy cover (CC), water cover (WC) and leaf litter (LL) on bird species richness and functional diversity (Fric, FEve, FDiv) of avian communities of the Midlands Mistbelts forest patches in the breeding and non-breeding season in KwaZulu-Natal, South Africa. The models are ranked by increasing ΔAIC. Top models ΔAIC < 2 are in bold.

| **Model** | **Explanatory variables** | **Significant variables (P-value)** | **AICc** | **ΔAIC** |
| --- | --- | --- | --- | --- |
| **Non Breeding** |  |  |  |  |
| Bird species Richness (Whole community) |  |  |  |  |
| **M0** | **TRIC+SC** | **TRIC (0.05); SC (0.00122**)** | **107.43** | **0.00** |
| **M1** | **TRIC+CC+SC** | **TRIC (0.02776*); SC (0.001***)** | **108.33** | **0.90** |
| **M2** | **SC** | **SC (2.83E-06***)** | **108.48** | **1.05** |
| **M3** | **CC*SC** | **SC (2.73E-06)** | **109.24** | **1.81** |
| **M4** | **TRIC+SC+WC** | **SC (0.00197***)** | **109.4** | **1.97** |
| **M5** | **WC*SC** | **WC (0.0810.); SC: WC (0.0608.)** | **109.4** | **1.97** |
| M6 | TRIC*SC | TRIC:SC (0.00572**) | 109.54 | 2.11 |
| M7 | Global Model | TRIC (0.0280); SC (0.0021**) | 110.32 | 2.89 |
| M8 | SC+CC | SC (2.76E-06***) | 110.32 | 2.89 |
| M9 | SC+WC | SC (9.27e-07***) | 110.46 | 3.03 |
| M10 | CC+SC+WC | SC (9.25E-06***) | 112.31 | 4.88 |
| M11 | TRIC | TRIC (0.000181***) | 113.52 | 6.09 |
| M12 | TRIC*WC | TRIC: WC (0.0658.) | 113.64 | 6.21 |
| M13 | TRIC+CC | TRIC (4.93E-05***) | 114.31 | 6.88 |
| M14 | TRIC+WC | TRIC (0.000343***) | 115.01 | 7.58 |
| M15 | TRIC*CC | TRIC (0.000543***) | 116.23 | 8.80 |
| M16 | Null Model | - | 119.79 | 12.36 |
| M17 | WC | - | 120.9 | 13.47 |
| M18 | CC | - | 121.73 | 14.30 |
| M19 | CC+WC | - | 122.73 | 15.30 |
| M20 | CC*WC | - | 124.62 | 17.19 |
| FRic (Whole community) |  |  |  |  |
| M0 | **SC** | - | **61.75** | **0.00** |
| M1 | **Null** | - | **61.90** | **0.15** |
| M2 | **TRIC** | - | **62.31** | **0.56** |
| M3 | **CC+SC** | - | **63.10** | **1.35** |
| M4 | **TRIC+CC** | - | **63.17** | **1.42** |
| M5 | **CC** | - | **63.46** | **1.71** |
| M6 | WC+SC | - | 63.75 | 2.00 |
| M7 | TRIC+WC | - | 64.24 | 2.49 |
| M8 | SC*CC | - | 64.36 | 2.61 |
| M9 | TRIC+CC+SC | - | 64.52 | 2.77 |
| M10 | TRIC*SC | - | 64.63 | 2.88 |
| M11 | CC+SC+WC | - | 65.09 | 3.34 |
| M12 | TRIC*CC | - | 65.10 | 3.35 |
| M14 | TRIC+CC+WC | - | 65.17 | 3.42 |
| M15 | CC+WC | - | 65.27 | 3.52 |
| M16 | SC*WC | - | 65.59 | 3.84 |
| M17 | TRIC*WC | - | 65.71 | 3.96 |
| M18 | Global | - | 66.49 | 4.74 |
| M19 | CC*WC | - | 67.00 | 5.25 |
| M20 | SC*WC*CC | - | 72.00 | 10.25 |
| Forest specialists |  |  |  |  |
| M0 | TRIC*SC | TRIC:SC (0.0866.) | **55.10** | **0.00** |
| M1 | SC | SC (2e-16***) | **55.48** | **0.38** |
| M2 | TRIC+SC | SC (0.0394*) | **56.56** | **1.46** |
| M3 | SC+WC | SC (0.0134*) | 57.43 | 2.33 |
| M4 | SC+CC | SC (0.00989**) | 57.48 | 2.38 |
| M5 | TRIC+CC+SC | SC (0.0414*) | 58.34 | 3.24 |
| M6 | WC*SC | - | 58.50 | 3.40 |
| M7 | CC*SC | SC (0.011*) | 59.40 | 4.30 |
| M8 | CC+SC+WC | SC (0.0148*) | 59.43 | 4.33 |
| M9 | TRIC | TRIC (0.0175*) | 59.53 | 4.43 |
| M10 | Global Model | - | 60.33 | 5.23 |
| M11 | TRIC*WC | - | 60.72 | 5.62 |
| M12 | TRIC+WC | TRIC (0.0259) | 60.89 | 5.79 |
| M13 | TRIC+CC | TRIC (0.0189*) | 61.24 | 6.14 |
| M14 | TRIC+SC+WC | - | 62.75 | 7.65 |
| M15 | TRIC*CC | TRIC (0.0194) | 62.96 | 7.86 |
| M16 | Null Model | - | 63.06 | 7.96 |
| M17 | WC | - | 63.70 | 8.60 |
| M18 | CC*WC | - | 64.49 | 9.39 |
| M19 | CC | - | 65.03 | 9.93 |
| M20 | SC*WC*CC | - | 65.54 | 10.44 |
|  | CC+WC | - | 65.57 | 10.47 |
| Forest generalists |  |  |  |  |
| M0 | SC+CC | - | **55.77** | **0.00** |
| M1 | SC | - | **55.89** | **0.12** |
| M2 | Null Model | - | **56.06** | **0.29** |
| M3 | TRIC+CC | - | **56.17** | **0.40** |
| M4 | CC*SC | - | **56.40** | **0.63** |
| M5 | TRIC | - | **56.84** | **1.07** |
| M6 | CC | - | **56.66** | **0.89** |
| M7 | TRIC+CC+SC | - | **57.09** | **1.32** |
| M8 | CC+SC+WC | - | **57.66** | **1.89** |
| M9 | TRIC+SC | - | 57.80 | 2.03 |
| M10 | WC | - | 57.86 | 2.09 |
| M11 | SC+WC | - | 57.89 | 2.12 |
| M12 | TRIC*SC | - | 57.96 | 2.19 |
| M13 | TRIC*CC | - | 58.04 | 2.27 |
| M14 | CC+WC | - | 58.56 | 2.79 |
| M15 | TRIC+WC | - | 58.79 | 3.02 |
| M16 | TRIC+SC+WC | - | 59.80 | 4.03 |
| M17 | WC*SC | - | 59.55 | 3.78 |
| M18 | Global Model | - | 58.91 | 3.14 |
| M19 | TRIC*WC | - | 60.15 | 4.38 |
| M20 | CC*WC | - | 60.26 | 4.49 |
| FEve (Whole community) |  |  |  |  |
| M0 | SC | - | **23.94** | **0.00** |
| M1 | TRIC | - | **23.99** | **0.05** |
| M2 | CC | - | **24.02** | **0.08** |
| M3 | Global Model | - | **24.06** | **0.12** |
| M4 | WC | - | **24.06** | **0.12** |
| M5 | SC+CC | - | **25.90** | **1.96** |
| M6 | SC+WC | - | **25.93** | **1.99** |
| M7 | TRIC+CC | - | **25.93** | **1.99** |
| M8 | TRIC+SC | - | 25.94 | 2.00 |
| M9 | TRIC+WC | - | 25.99 | 2.05 |
| M10 | CC+WC | - | 26.02 | 2.08 |
| M11 | CC*SC | - | 27.81 | 3.87 |
| M12 | TRIC*CC | - | 27.86 | 3.92 |
| M13 | TRIC+CC+SC | - | 27.90 | 3.96 |
| M14 | CC+SC+WC | - | 27.90 | 3.96 |
| M15 | WC*SC | - | 27.94 | 4.00 |
| M16 | TRIC+SC+WC | - | 27.94 | 4.00 |
| M17 | TRIC*SC | - | 27.94 | 4.00 |
| M18 | TRIC*WC | - | 27.98 | 4.04 |
| M19 | CC*WC | - | 27.99 | 4.05 |
| M20 | Null Model | - | 29.89 | 5.95 |
| Forest specialists |  |  |  |  |
| M0 | SC | SC (0.0254*) | **21.05** | **0.00** |
| M1 | TRIC | - | **22.63** | **1.58** |
| M2 | WC | - | **22.89** | **1.84** |
| M3 | CC | - | **22.92** | **1.87** |
| M4 | SC+CC | SC (0.0144*) | 24.04 | 2.99 |
| M5 | SC+WC | SC (0.0196*) | 24.04 | 2.99 |
| M6 | TRIC+SC+WC | SC (0.0158*) | 26.04 | 4.99 |
| M7 | CC+SC+WC | SC (0.0428*) | 26.04 | 4.99 |
| M8 | TRIC+SC | SC (0.0193*) | 24.04 | 2.99 |
| M9 | TRIC+WC | - | 24.61 | 3.56 |
| M10 | TRIC+CC | SC (0.0187*) | 24.62 | 3.57 |
| M11 | CC+WC | - | 24.85 | 3.80 |
| M12 | TRIC*SC | SC (0.0279*) | 25.58 | 4.53 |
| M13 | Null Model | - | 25.92 | 4.87 |
| M14 | WC*SC | SC (0.0250*) | 25.97 | 4.92 |
| M15 | Global Model | SC (0.0119*) | 28.03 | 6.98 |
| M16 | TRIC+CC+SC | SC (0.0174*) | 26.03 | 4.98 |
| M17 | CC*SC | SC (0.0249*) | 26.04 | 4.99 |
| M18 | TRIC*CC | - | 26.23 | 5.18 |
| M19 | TRIC*WC | - | 26.23 | 5.18 |
| M20 | CC*WC | - | 26.85 | 5.80 |
| Forest generalists |  |  |  |  |
| M0 | CC | CC (0.0127*) | **22.18** | **0.00** |
| M1 | SC | - | **23.25** | **1.07** |
| M2 | TRIC | - | **23.37** | **1.19** |
| M3 | WC | - | **23.39** | **1.21** |
| M4 | SC+CC | CC (0.0190*) | 24.75 | 2.57 |
| M5 | TRIC+SC | - | 25.07 | 2.89 |
| M6 | TRIC+CC | CC (0.0415*) | 25.07 | 2.89 |
| M7 | SC+WC | - | 25.11 | 2.93 |
| M8 | CC+WC | CC (0.0104*) | 25.17 | 2.99 |
| M9 | TRIC+WC | - | 25.37 | 3.19 |
| M10 | Null Model | - | 26.40 | 4.22 |
| M11 | CC*SC | CC:SC (0.0413*) | 26.70 | 4.52 |
| M12 | CC+SC+WC | - | 26.72 | 4.54 |
| M13 | TRIC+CC+SC | CC (0.0136*) | 26.76 | 4.58 |
| M14 | TRIC*SC | - | 26.95 | 4.77 |
| M15 | TRIC*CC | CC (0.0203*) | 27.07 | 4.89 |
| M16 | TRIC+SC+WC | - | 27.07 | 4.89 |
| M17 | WC*SC | - | 27.11 | 4.93 |
| M18 | CC*WC | CC (0.0397*) | 27.13 | 4.95 |
| M19 | TRIC*WC | - | 27.21 | 5.03 |
| M20 | Global Model | CC (0.0177*) | 28.72 | 6.54 |
| FDiv (Whole community) |  |  |  |  |
| M0 | Null Model |  | **29.99** | **0.00** |
| M1 | TRIC | - | **31.97** | **1.98** |
| M2 | SC | - | 31.99 | 2.00 |
| M3 | WC | - | 31.99 | 2.00 |
| M4 | CC |  | 31.99 | 2.00 |
| M5 | TRIC+SC | - | 33.98 | 3.99 |
| M6 | TRIC+CC | - | 33.98 | 3.99 |
| M7 | TRIC+WC | - | 33.98 | 3.99 |
| M8 | CC+WC | - | 33.99 | 4.00 |
| M9 | SC+CC | - | 33.99 | 4.00 |
| M10 | SC+WC | - | 33.99 | 4.00 |
| M11 | TRIC*CC | - | 35.96 | 5.97 |
| M12 | TRIC+CC+SC | - | 35.97 | 5.98 |
| M13 | TRIC*WC | - | 35.97 | 5.98 |
| M14 | TRIC*SC | - | 35.98 | 5.99 |
| M15 | CC*SC | - | 35.98 | 5.99 |
| M16 | TRIC+SC+WC | - | 35.98 | 5.99 |
| M17 | CC*WC | - | 35.99 | 6.00 |
| M18 | WC*SC | - | 35.99 | 6.00 |
| M19 | CC+SC+WC | - | 35.99 | 6.00 |
| M20 | Global Model |  | 37.98 | 7.99 |
| Forest specialists |  |  |  |  |
| M0 | SC | SC (0.00039***) | **31.64** | **0.00** |
| M1 | TRIC | - | **32.00** | **0.36** |
| M2 | CC | - | **32.10** | **0.46** |
| M3 | WC | - | **32.22** | **0.58** |
| M4 | Null Model | - | **32.26** | **0.62** |
| M5 | SC+CC | SC (0.00046***) | **33.37** | **1.73** |
| M6 | SC+WC | - | 33.64 | 2.00 |
| M7 | TRIC+SC | SC (0.00047***) | 33.64 | 2.00 |
| M8 | TRIC+CC | - | 33.66 | 2.02 |
| M9 | TRIC+WC | - | 33.99 | 2.35 |
| M10 | CC+WC | - | 34.08 | 2.44 |
| M11 | CC*SC | SC (0.00019***) | 35.05 | 3.41 |
| M12 | TRIC+CC+SC | SC (0.00034***) | 35.31 | 3.67 |
| M13 | TRIC*SC | SC (0.00017***) | 35.33 | 3.69 |
| M14 | CC+SC+WC | SC (0.00022***) | 35.34 | 3.70 |
| M15 | WC*SC | - | 35.61 | 3.97 |
| M16 | TRIC*CC | - | 35.63 | 3.99 |
| M17 | TRIC+SC+WC | SC (0.00051***) | 35.64 | 4.00 |
| M18 | TRIC*WC | - | 35.74 | 4.10 |
| M19 | CC*WC | - | 36.02 | 4.38 |
| M20 | Global Model | SC (0.00012***) | 37.27 | 5.63 |
| Forest generalists |  |  |  |  |
| **M0** | **SC** | **-** | **29.17** | **0.00** |
| **M1** | **CC** | **CC (0.0010**)** | **29.17** | **0.00** |
| **M2** | **WC** | **-** | **29.41** | **0.24** |
| **M4** | **TRIC** | **-** | **29.36** | **0.19** |
| **M5** | **SC+CC** | **CC (0.0031**)** | **30.77** | **1.60** |
| **M6** | **TRIC+CC** | **CC (0.0014**)** | **30.93** | **1.76** |
| **M7** | TRIC+SC | **-** | 31.17 | 2.00 |
| **M8** | SC+WC | **-** | 31.17 | 2.00 |
| M9 | TRIC+WC | - | 31.34 | 2.17 |
| M10 | TRIC+CC+SC | CC (0.00174**) | 32.73 | 3.56 |
| M11 | CC+SC+WC | CC (0.0011**) | 32.76 | 3.59 |
| M12 | TRIC*CC | - | 32.84 | 3.67 |
| M13 | CC*SC | CC:SC (0.0013**) | 32.99 | 3.82 |
| M15 | TRIC*SC | - | 33.02 | 3.85 |
| M16 | CC*WC | - | 33.09 | 3.92 |
| M17 | WC*SC | - | 33.15 | 3.98 |
| M18 | TRIC+SC+WC | - | 33.17 | 4.00 |
| M19 | TRIC*WC | - | 33.22 | 4.05 |
| M20 | Global Model | CC (0.00321**) | 34.72 | 5.55 |
| **Breeding** |  |  |  |  |
| Bird species richness |  |  |  |  |
| M0 | TRIC | TRIC (2.91E-06***) | **109.2** | **0.00** |
| M1 | TRIC+CC | TRIC (8.86E-07***) | **109.33** | **0.13** |
| M2 | TRIC*CC | TRIC (1.65E-05***) | **110.03** | **0.83** |
| M3 | TRIC*LL | TRIC (0.08221.); TRIC:LL (0.00658**) | **110.14** | **0.94** |
| M4 | TRIC+CC+SC | TRIC (1.77e-05***) | **110.52** | **1.32** |
| M5 | TRIC+SC | TRIC (2e-16***) | **110.79** | **1.59** |
| M6 | TRIC+LL | TRIC (3.89E-05***) | **110.8** | **1.60** |
| M7 | TRIC+SC+LL | TRIC (0.00157**) | 111.92 | 2.72 |
| M8 | TRIC*SC | TRIC (2.65e-05***) | 111.93 | 2.73 |
| M9 | Global Model | TRIC (0.00048***) | 112.22 | 3.02 |
| M10 | SC+LL | SC (0.0219*); LL (0.0365*) | 117.36 | 8.16 |
| M11 | CC*SC | CC:SC (0.0465*) | 118.5 | 9.30 |
| M12 | CC+SC+LL | SC (0.0172*); LL (0.0664.) | 118.87 | 9.67 |
| M13 | SC | SC (0.0663.) | 119.09 | 9.89 |
| M14 | LL*SC | LL (0.0361*); SC (0.0410) | 119.32 | 10.12 |
| M15 | LL | - | 119.74 | 10.54 |
| M16 | SC+CC | SC (0.0376*) | 119.83 | 10.63 |
| M17 | Null Model |  | 119.86 | 10.66 |
| M18 | CC | - | 121.4 | 12.20 |
| M19 | CC+LL | - | 121.6 | 12.40 |
| M20 | CC*LL | LL (0.0996) | 122.11 | 12.91 |
| Fric (Whole community) |  |  |  |  |
| **M0** | **TRIC** | **-** | **60.60** | **0.00** |
| **M1** | **LL** | **-** | **61.04** | **0.44** |
| **M2** | **CC** | **-** | **61.11** | **0.51** |
| **M3** | **SC** | **-** | **61.12** | **0.52** |
| **M4** | **Null Model** | **-** | **61.12** | **0.52** |
| **M5** | **TRIC+SC** | **-** | **62.49** | **1.89** |
| **M6** | **TRIC+CC** | **-** | **62.58** | **1.98** |
| **M7** | TRIC+LL |  | 62.60 | 2.00 |
| M8 | CC+LL | - | 62.98 | 2.38 |
| M9 | SC+LL | - | 63.04 | 2.44 |
| M10 | SC+CC | - | 63.11 | 2.51 |
| M11 | TRIC+CC+SC | - | 64.46 | 3.86 |
| M12 | TRIC*SC | - | 64.47 | 3.87 |
| M13 | TRIC*CC | - | 64.52 | 3.92 |
| M14 | TRIC*LL | - | 64.52 | 3.92 |
| M15 | TRIC+SC+LL | - | 64.58 | 3.98 |
| M16 | CC*SC | - | 64.70 | 4.10 |
| M17 | CC+SC+LL | - | 64.97 | 4.37 |
| M18 | CC*LL | - | 64.98 | 4.38 |
| M19 | LL*SC | - | 65.02 | 4.42 |
| M20 | Global Model | - | 66.46 | 0.00 |
| Forest specialists |  |  |  |  |
| M0 | **TRIC** | **-** | **56.94** | **0.00** |
| M2 | **LL** | **-** | **58.47** | **1.53** |
| M3 | **TRIC+LL** | **-** | **58.81** | **1.87** |
| M4 | **TRIC+SC** | **-** | **58.81** | **1.87** |
| M5 | **TRIC+CC** | **-** | **58.87** | **1.93** |
| M6 | SC | - | 59.24 | 2.30 |
| M7 | CC | - | 59.30 | 2.36 |
| M8 | TRIC*LL | - | 59.92 | 2.98 |
| M9 | SC+LL | - | 60.28 | 3.34 |
| M10 | CC+LL | - | 60.47 | 3.53 |
| M11 | TRIC+SC+LL | - | 60.74 | 3.80 |
| M12 | TRIC*SC | - | 60.76 | 3.82 |
| M13 | TRIC+CC+SC | - | 60.76 | 3.82 |
| M14 | TRIC*CC | - | 60.87 | 3.93 |
| M15 | SC+CC | - | 61.18 | 4.24 |
| M16 | CC*SC | - | 61.50 | 4.56 |
| M17 | CC+SC+LL | - | 62.27 | 5.33 |
| M18 | LL*SC | - | 62.28 | 5.34 |
| M19 | CC*LL | - | 62.37 | 5.43 |
| M20 | Global Model | - | 62.72 | 5.78 |
| Forest generalists |  |  |  |  |
| M0 | **TRIC** |  | **55.00** | **0.00** |
| M1 | **LL** |  | **55.98** | **0.98** |
| M2 | **CC** |  | **56.00** | **1.00** |
| M3 | **SC** |  | **56.26** | **1.26** |
| M4 | **TRIC*CC** |  | **56.69** | **1.69** |
| M5 | **TRIC+CC** |  | **56.73** | **1.73** |
| M6 | **TRIC+SC** |  | **56.77** | **1.77** |
| M7 | TRIC+LL |  | 57.00 | 2.00 |
| M8 | CC+LL |  | 57.84 | 2.84 |
| M9 | SC+LL |  | 57.97 | 2.97 |
| M10 | SC+CC |  | 58.00 | 3.00 |
| M11 | TRIC+CC+SC |  | 58.54 | 3.54 |
| M12 | TRIC*SC |  | 58.75 | 3.75 |
| M13 | TRIC+SC+LL |  | 58.78 | 3.78 |
| M14 | TRIC*LL |  | 58.99 | 3.99 |
| M15 | Null Model |  | 59.26 | 4.26 |
| M16 | CC*SC |  | 59.80 | 4.80 |
| M17 | CC+SC+LL |  | 59.83 | 4.83 |
| M18 | CC*LL |  | 59.83 | 4.83 |
| M19 | LL*SC |  | 59.90 | 4.90 |
| M20 | Global Model |  | 60.49 | 5.49 |
| FEve |  |  |  |  |
| Whole community |  |  |  |  |
| M0 | TRIC | - | **25.33** | **0.00** |
| M1 | SC | - | **25.42** | **0.09** |
| M2 | LL | - | **25.42** | **0.09** |
| M3 | CC | - | **25.44** | **0.11** |
| M4 | Null Model | - | **26.44** | **1.11** |
| M5 | TRIC+CC | - | 27.33 | 2.00 |
| M6 | TRIC+LL | - | 27.33 | 2.00 |
| M7 | TRIC+SC | - | 27.33 | 2.00 |
| M8 | SC+LL | - | 27.39 | 2.06 |
| M9 | CC+LL | - | 27.41 | 2.08 |
| M10 | SC+CC | - | 27.42 | 2.09 |
| M11 | TRIC*LL | - | 29.31 | 3.98 |
| M12 | TRIC*SC | - | 29.32 | 3.99 |
| M13 | TRIC+CC+SC | - | 29.33 | 4.00 |
| M14 | TRIC+SC+LL | - | 29.33 | 4.00 |
| M15 | TRIC*CC | - | 29.36 | 4.03 |
| M16 | LL*SC | - | 29.37 | 4.04 |
| M17 | CC+SC+LL | - | 29.38 | 4.05 |
| M18 | CC*SC | - | 29.38 | 4.05 |
| M19 | CC*LL | - | 29.39 | 4.06 |
| M20 | Global Model | - | 31.35 | 6.02 |
| Forest specialists |  |  |  |  |
| M0 | TRIC | TRIC (0.0239*) | **23.43** | **0.00** |
| M1 | LL | - | **23.62** | **0.19** |
| M2 | CC | - | **23.74** | **0.31** |
| M3 | SC | - | **23.83** | **0.40** |
| M4 | SC+LL | - | **25.23** | **1.80** |
| M5 | TRIC+CC | TRIC (0.032*) | **25.25** | **1.82** |
| M6 | TRIC+LL | TRIC (0.0327*) | **25.35** | **1.92** |
| M7 | TRIC+SC | TRIC (0.0349*) | 25.43 | 2.00 |
| M8 | CC+LL | - | 25.57 | 2.14 |
| M9 | SC+CC | - | 25.69 | 2.26 |
| M10 | TRIC*LL | TRIC (0.0635.) | 26.98 | 3.55 |
| M11 | CC*SC | - | 27.11 | 3.68 |
| M12 | TRIC*CC | TRIC (0.0261*) | 27.25 | 3.82 |
| M13 | TRIC+CC+SC | TRIC (0.0253*) | 27.26 | 3.83 |
| M14 | TRIC*SC | TRIC (0.0409*) | 27.26 | 3.83 |
| M15 | TRIC+SC+LL | TRIC (0.0425*) | 27.35 | 3.92 |
| M16 | CC+SC+LL | - | 27.46 | 4.03 |
| M17 | LL*SC | - | 27.51 | 4.08 |
| M18 | CC*LL | - | 27.56 | 4.13 |
| M19 | Null Model |  | 27.87 | 4.44 |
| M20 | Global Model | TRIC (0.0259*) | 29.56 | 6.13 |
| Forest generalists |  |  |  |  |
| M0 | TRIC | TRIC (0.00045***) | **25.15** | 0.00 |
| M1 | SC |  | **25.27** | 0.12 |
| M2 | LL | LL (0.00052***) | **25.30** | 0.15 |
| M3 | CC |  | **25.30** | 0.15 |
| M4 | Null Model |  | **26.30** | 1.15 |
| M5 | TRIC+LL | LL (0.00042***) | **27.11** | 1.96 |
| M6 | TRIC+CC | TRIC (0.00062***) | **27.14** | 1.99 |
| M7 | TRIC+SC | TRIC (0.00030**) | **27.15** | 2.00 |
| M8 | SC+CC |  | 27.27 | 2.12 |
| M9 | SC+LL | LL (0.00016***) | 27.27 | 2.12 |
| M10 | CC+LL | LL (0.0009***) | 27.3 | 2.15 |
| M11 | TRIC+CC+SC | TRIC (0.00081***) | 29.14 | 3.99 |
| M12 | TRIC*CC | TRIC (0.00069***) | 29.14 | 3.99 |
| M13 | TRIC*LL | TRIC:LL (0.00012***) | 29.14 | 3.99 |
| M14 | TRIC*SC |  | 29.14 | 3.99 |
| M15 | CC*SC | CC (0.0045**) | 29.22 | 4.07 |
| M16 | CC*LL | LL:CC (0.00021***) | 29.25 | 4.10 |
| M17 | LL*SC | LL (0.0009**8) | 29.26 | 4.11 |
| M18 | CC+SC+LL | LL (0.00033***); CC (0.0014**) | 29.26 | 4.11 |
| M19 | TRIC+SC+LL | LL (0.00061***) | 29.22 | 4.07 |
| M20 | Global Model | TRIC (0.00042***); LL (0.00015***) | 31.11 | 5.96 |
| FDiv |  |  |  |  |
| Whole community |  |  |  |  |
| M0 | TRIC | - | **31.91** | **0.00** |
| M1 | CC | - | **31.91** | **0.00** |
| M2 | LL | - | **31.91** | **0.00** |
| M3 | SC | - | **31.92** | **0.01** |
| M4 | TRIC+SC | - | 33.91 | 2.00 |
| M5 | TRIC+CC | - | 33.91 | 2.00 |
| M6 | SC+CC | - | 33.91 | 2.00 |
| M7 | SC+LL | - | 33.91 | 2.00 |
| M8 | CC+LL | - | 33.91 | 2.00 |
| M9 | TRIC+LL | - | 33.91 | 2.00 |
| M10 | LL*SC | - | 35.90 | 3.99 |
| M11 | TRIC*CC | - | 35.90 | 3.99 |
| M12 | TRIC*LL | - | 35.91 | 4.00 |
| M13 | TRIC*SC | - | 35.91 | 4.00 |
| M14 | CC*LL | - | 35.91 | 4.00 |
| M15 | CC*SC | - | 35.91 | 4.00 |
| M16 | TRIC+CC+SC | - | 35.91 | 4.00 |
| M17 | TRIC+SC+LL | - | 35.91 | 4.00 |
| M18 | CC+SC+LL | - | 35.91 | 4.00 |
| M19 | Null Model | - |  |  |
| M20 | Global Model | - |  |  |
| Forest specialists |  |  |  |  |
| M1 | TRIC | TRIC (0.00136**) | **32.58** | **0.00** |
| M2 | SC | SC (0.0156*) | **32.58** | **0.00** |
| M3 | LL | - | **33.56** | **0.98** |
| M4 | CC+LL | - | **34.56** | **1.98** |
| M5 | TRIC+CC | TRIC (0.0064**) | **34.56** | **1.98** |
| M6 | TRIC+SC | TRIC (0.0023**); SC (0.0340*) | **34.57** | **1.99** |
| M7 | TRIC+LL |  | **34.57** | **1.99** |
| M8 | SC+CC | SC (0.0145*) | 34.58 | 2.00 |
| M9 | SC+LL | SC (0.0134*) | 34.58 | 2.00 |
| M10 | TRIC*SC | TRIC (0.00321**) | 35.57 | 2.99 |
| M11 | Null Model | - | 35.61 | 3.03 |
| M12 | TRIC*CC | TRIC (0.0045**) | 36.56 | 3.98 |
| M13 | TRIC*LL | TRIC (0.0045**) | 36.56 | 3.98 |
| M14 | TRIC+CC+SC | SC (0.0437*) | 36.56 | 3.98 |
| M15 | CC*LL | - | 36.57 | 3.99 |
| M16 | CC*SC | - | 36.57 | 3.99 |
| M17 | CC+SC+LL | SC (0.0183*) | 36.57 | 3.99 |
| M18 | TRIC+SC+LL | TRIC (0.0018**); SC (0.0414*) | 36.57 | 3.99 |
| M19 | LL*SC | - | 36.58 | 4.00 |
| M20 | Global Model | TRIC (0.0071**); SC (0.0304*) | 38.56 | 5.98 |
| Forest generalists |  |  |  |  |
| M0 | TRIC | - | **30.24** | **0.00** |
| M1 | SC | SC (0.0169*) | **30.24** | **0.00** |
| M2 | CC | - | **30.26** | **0.02** |
| M3 | LL | - | **30.26** | **0.02** |
| M4 | SC+CC | SC (0.0243*) | **32.21** | **1.97** |
| M5 | SC+LL | SC (0.0268*) | **32.22** | **1.98** |
| M6 | TRIC+CC | - | **32.23** | **1.99** |
| M7 | TRIC+SC | SC (0.0416*) | 32.24 | 2.00 |
| M8 | CC+LL | - | 32.24 | 2.00 |
| M9 | TRIC+LL | - | 32.25 | 2.01 |
| M10 | TRIC*CC | - | 34.19 | 3.95 |
| M11 | CC*SC | SC (0.0413*) | 34.19 | 3.95 |
| M12 | CC+SC+LL | SC (0.0119*) | 34.19 | 3.95 |
| M13 | CC*LL | - | 34.20 | 3.96 |
| M14 | TRIC*SC | SC (0.0167*) | 34.20 | 3.96 |
| M15 | TRIC+CC+SC | SC (0.0149*) | 34.21 | 3.97 |
| M16 | LL*SC | SC (0.0128*) | 34.22 | 3.98 |
| M17 | TRIC+SC+LL | SC (0.04218) | 34.22 | 3.98 |
| M18 | TRIC*LL | - | 34.23 | 3.99 |
| M19 | Global Model | SC (0.0314*) | 36.20 | 5.96 |
| M20 | Null Model | - | 38.28 | 8.04 |

**Supplementary Information Table S3** Tree species in 14 selected Mistbelt forest patches of the Midlands of KwaZulu-Natal, South Africa.

| **Family** | **Scientific name** | **Common name** |
| --- | --- | --- |
| Anacardiaceae | *Searsia chirindensis* | Red currant |
| Apocynaceae | *Carissa bispinosa* | Num-num |
|  | *Strophanthus speciosus* | Forest poison-rope |
| Aquifoliaceae | *Ilex mitis* | Cape holly |
| Araliaceae | *Cussonia sphaerocephala* | Forest cabbage-tree |
| Buddlejaceae | *Nuxia floribunda* | Forest-elder |
| Celastraceae | *Elaeodendron croceum*^D^ | Common saffron |
|  | *Gymnosporia harveyana* | Round-fruit forest spikethorn |
|  | *Pterocelastrus rostratus*^D^ | Red candlewood |
|  | *Salacia gerrardii* | Forest lemon-rope |
| Celtidaceae | *Celtis africana* | White-stinkwood |
|  | *Trema orientalis* | Pigeonwood |
|  | *Trimeria grandifolia* | Wild-mulberry |
| Combretaceae | *Combretum edwardsii* | Forest climbing bushwillow |
| Connaraceae | *Cnestis polyphylla* | Itch-pod |
| Ebenaceae | *Diospyros whyteana* | Bladder-nut |
| Euphorbiaceae | *Drypetes gerrardii* | Forest ironplum |
|  | *Micrococca capensis* | False bead-string |
| Fabaceae | *Calpurnia aurea* | Wild laburnum |
|  | *Dalbergia obovata* | Climbing flat-bean |
| Flacourtiaceae | *Dovyalis lucida* | Glossy Kei-apple |
|  | *Dovyalis rhamnoides* | Sourberry Kei-apple |
|  | *Kiggelaria africana* | Wild-peach |
|  | *Scolopia flanaganii* | Kei thorn-pear |
|  | *Scolopia mundii* | Red thorn-pear |
|  | *Scolopia zeyheri* | Thorn-pear |
| Icacinaceae | *Apodytes dimidiata* | White-pear |
|  | *Cassinopsis illicifolia* | Lemon-thorn |
| Lamiaceae | *Clerodendrum glabrum* | Cats-whiskers |
| Lauraceae | *Cryptocarya mrtifolia*^V^ | Myrtle wild-quince |
|  | *Cryptocarya woodii*^D^ | River wild-quince |
|  | *Ocotea bullatta*^E^ | Stinkwood |
| Meliaceae | *Ekebergia capensis* | Cape-ash |
|  | *Trichilia dregeana* | Forest Natal mahogany |
| Monimiaceae | *Xymalos monospora* | Lemonwood |
| Moraceae | *Ficus craterostoma* | Forest fig |
| Myrsinaceae | *Rapanea melanophloes*^D^ | Cape-beech |
| Myrtaceae | *Eugenia zuluensis* | Paperbark myrtle |
|  | *Syzigium gerrrardii* | Forest umdoni |
| Ochnaceae | *Ochna arborea* | - |
| Oleaceae | *Chionanthus foveolatus* | Pock-ironwood |
|  | *Chionanthus pelglerae* | Giant pock-ironwood |
|  | *Olea capensis* | Ironwood |
| Pittosporaceae | *Pittosporum viridiflorum*^P^ | Cheesewood |
| Podocarpaceae | *Afrocarpus falcatus*^P^ | Common yellowwood |
|  | *Podocarpus henkelii*^P^ | Dropping-leaf yellowwood |
|  | *Podocarpus latifolia*^P^ | Broad-leaf yellowwood |
| Ptaeroxylaceae | *Ptaeroxylon obliquum* | Sneezewood |
| Rhamnaceae | *Scutia myrtina* | Cat-thorn |
| Rhizophoraceae | *Cassipourea gummiflua*^V^ | Large-leaf onionwood |
|  | *Cassipourea malosana*^D^ | Onionwood |
| Rosaceae | *Prunus africana*^V^ | Red-stinkwood |
| Rubiaceae | *Canthium ciliatum* | Hairy turkey-berry |
|  | *Canthium kuntzeanum* | Mountain turkey-berry |
|  | *Hypecanthus amoenus* | Spiny-gardenia |
|  | *Tricalysia lanceolata*^E^ | Jackal-cofee |
| Rutaceae | *Calodendrum capense* | Cape-chestnut |
|  | *Clausena anisata* | Horsewood |
|  | *Vepris lanceolata* | White-ironwood |
|  | *Zanthoxylum capense* | Small knobwood |
|  | *Zanthoxylum dayvi* | Forest knobwood |
| Sapindaceae | *Allophylus africanus* | African false-currant |
|  | *Allophylus dregeanus* | Simple-leaf False-currant |
| Scrophulariaceae | *Halleria lucida* | Tree fuchsia |
| Solanaceae | *Solanum giganteum* | Healing-leaf tree |
|  | **Solanum mauritianum* | Bugweed |
| Thymeleaceae | *Dais cotinifolia* | Pompon tree |
|  | *Peddiea africana* | Poison-olive |

*Alien invasive

^D^ Declining

^E^Endangered

^P^Protected

^V^Vulnerable

**Supplementary Information Table S4** Tree species presence and absence in selected forest 14 patches in the Midlands of KwaZulu-Natal, South Africa.

|  | **Forest Patch** | | | | | | | | | | | | | |
| --- | --- | --- | --- | --- | --- | --- | --- | --- | --- | --- | --- | --- | --- | --- |
| **Region** | **Karkloof** | | | | **Balgowan** | | **Dargle** | | | | **Bulwer** | | | |
| **Tree species** |  | | | |  | |  | | | |  | | | |
|  | 1 | 2 | 3 | 4 | 5 | 6 | 7 | 8 | 9 | 10 | 11 | 12 | 13 | 14 |
| *Searsia chirindensis* | 🗸 | 🗸 | - | - | - | - | - | - | - | - | 🗸 | - | - |  |
| *Carissa bispinosa* | 🗸 | 🗸 | 🗸 | 🗸 | 🗸 | 🗸 | 🗸 | 🗸 | - | - | 🗸 | 🗸 | 🗸 | 🗸 |
| *Strophanthus speciosus* | 🗸 | 🗸 | - | - | - | - | - | - | - | - | - | - | - | 🗸 |
| *Ilex mitis* | 🗸 | 🗸 | - | 🗸 | - | - | - | - | - | - | - | - | - | - |
| *Cussonia sphaerocephala* | 🗸 | 🗸 | 🗸 | 🗸 | - | - | 🗸 | - | - | - | 🗸 | 🗸 | 🗸 | 🗸 |
| *Nuxia floribunda* | 🗸 | 🗸 | - | - | - | - | - | - | - | - | - | - | - | - |
| *Elaeodendron croceum*^D^ | 🗸 | 🗸 | 🗸 | 🗸 | 🗸 | 🗸 | - | - | - | - | 🗸 | - | 🗸 | 🗸 |
| *Gymnosporia harveyana* | 🗸 | 🗸 | 🗸 | 🗸 | 🗸 | 🗸 | 🗸 | 🗸 | 🗸 | 🗸 | 🗸 | 🗸 | 🗸 | 🗸 |
| *Pterocelastrus rostratus*^D^ | - | - | - | - | - | - | - | - | - | - | 🗸 | - | - | 🗸 |
| *Salacia gerrardii* | 🗸 | 🗸 | 🗸 | 🗸 | 🗸 | 🗸 | 🗸 | - | - | - | - | 🗸 | 🗸 | 🗸 |
| *Celtis africana* | 🗸 | 🗸 | 🗸 | 🗸 | - | - | 🗸 | - | - | - | 🗸 | 🗸 | 🗸 | 🗸 |
| *Trema orientalis* | 🗸 | 🗸 | - | - | - | - | - | - | - | - | - | - | - | - |
| *Trimeria grandifolia* | 🗸 | 🗸 | 🗸 | 🗸 | 🗸 | 🗸 | - | - | 🗸 | - | 🗸 | 🗸 | 🗸 | 🗸 |
| *Combretum edwardsii* | 🗸 | 🗸 | - | - | - | - | - | - | - | - | - | - | 🗸 | - |
| *Cnestis polyphylla* | 🗸 | 🗸 | 🗸 | **-** | - | - | - | - | - | - | - | - | - | - |
| *Diospyros whyteana* | 🗸 | 🗸 | 🗸 | 🗸 | 🗸 | 🗸 | 🗸 | 🗸 | - | - | 🗸 | 🗸 | 🗸 | 🗸 |
| *Drypetes gerrardii* | - | 🗸 | - | - | 🗸 | 🗸 | 🗸 | 🗸 | - | - | 🗸 | - | 🗸 | - |
| *Micrococca capensis* | 🗸 | 🗸 | 🗸 | **-** | - | - | - | - | - | - | - | - | - | - |
| *Calpurnia aurea* | 🗸 | 🗸 | 🗸 | 🗸 | - | - | - | - | - | - | 🗸 | 🗸 | 🗸 | 🗸 |
| *Dalbergia obovata* | 🗸 | 🗸 | 🗸 | - | - | - | - | - | - | - | - | - | - | - |
| *Dovyalis lucida* | 🗸 | 🗸 | - | - | - | - | - | - | - | - | 🗸 | - | - | - |
| *Dovyalis rhamnoides* | 🗸 | 🗸 | - | - | - | - | - | - | - | - | 🗸 | - | 🗸 | - |
| *Kiggelaria africana* | 🗸 | 🗸 | 🗸 | 🗸 | 🗸 | 🗸 | 🗸 | 🗸 | - | - | 🗸 | 🗸 | 🗸 | 🗸 |
| *Scolopia flanaganii* | 🗸 | 🗸 | - | - | 🗸 | 🗸 | - | - | - | - | 🗸 | - | - | 🗸 |
| *Scolopia mundii* | - | - | - | - | - | - | - | - | - | - | - | - | 🗸 | 🗸 |
| *Scolopia zeyheri* | 🗸 | 🗸 | - | - | 🗸 | 🗸 | 🗸 | 🗸 | - | - | 🗸 | 🗸 | 🗸 | - |
| *Apodytes dimidiata* | **-** | **-** | **-** | **-** | **-** | **-** | **-** | **-** | **-** | **-** | 🗸 | **-** | **-** | **-** |
| *Cassinopsis illicifolia* | 🗸 | 🗸 | 🗸 | 🗸 | 🗸 | 🗸 | 🗸 | - | - | - | - | 🗸 | 🗸 | - |
| *Clerodendrum glabrum* | **-** | **-** | **-** | **-** | **-** | **-** | **-** | **-** | **-** | **-** | **-** | **-** | 🗸 | **-** |
| *Cryptocarya mrtifolia*^V^ | 🗸 | 🗸 | - | 🗸 | - | - | - | - | - | - | - | - | - | - |
| *Cryptocarya woodii*^D^ | 🗸 | 🗸 | 🗸 | 🗸 | 🗸 | 🗸 | 🗸 | - | 🗸 | 🗸 | 🗸 | 🗸 | 🗸 | - |
| *Ocotea bullatta*^E^ | 🗸 | - | - | - | - | 🗸 | - | - | - | - | - | - | 🗸 | - |
| *Ekebergia capensis* | 🗸 | 🗸 | - | - | - | - | - | - | - | - | - | - | - | - |
| *Trichilia dregeana* | 🗸 | 🗸 | - | - | - | - | - | - | - | - | - | - | - | - |
| *Xymalos monospora* | 🗸 | 🗸 | 🗸 | 🗸 | 🗸 | 🗸 | 🗸 | 🗸 | - | - | - | 🗸 | 🗸 | 🗸 |
| *Ficus craterostoma* | 🗸 | 🗸 | 🗸 | 🗸 | 🗸 | 🗸 | 🗸 | - | - | - | 🗸 | - | 🗸 | - |
| *Rapanea melanophloes*^D^ | 🗸 | 🗸 | 🗸 | 🗸 | 🗸 | 🗸 | 🗸 | 🗸 | - | - | - | - | 🗸 | 🗸 |
| *Eugenia zuluensis* | 🗸 | 🗸 | 🗸 | 🗸 | 🗸 | 🗸 | 🗸 | **-** | 🗸 | 🗸 | 🗸 | 🗸 | 🗸 | 🗸 |
| *Syzigium gerrrardii* | 🗸 | 🗸 | 🗸 | 🗸 | - | - | - | - | - | - | - | - | - | - |
| *Ochna arborea* | 🗸 | 🗸 | 🗸 | 🗸 | - | - | - | 🗸 | - | - | - | - | 🗸 | - |
| *Chionanthus foveolatus* | - | - | - | - | - | - | - | - | - | - | 🗸 | - | - | - |
| *Chionanthus pelglerae* | **-** | 🗸 | - | - | - | - | - | - | - | - | - | - | - | - |
| *Olea capensis* | 🗸 | 🗸 | - | - | - | - | 🗸 | - | - | - | - | - | - | - |
| *Pittosporum viridiflorum*^P^ | - | - | - | - | - | - | 🗸 | - | - | - | 🗸 | - | 🗸 | 🗸 |
| *Afrocarpus falcatus*^P^ | 🗸 | 🗸 | 🗸 | 🗸 | 🗸 | 🗸 | 🗸 | 🗸 | 🗸 | **-** | 🗸 | 🗸 | 🗸 | 🗸 |
| *Podocarpus henkelii*^P^ | 🗸 | 🗸 | 🗸 | **-** | 🗸 | 🗸 | 🗸 | **-** | **-** | **-** | 🗸 | 🗸 | 🗸 | 🗸 |
| *Podocarpus latifolia*^P^ | 🗸 | 🗸 | 🗸 | 🗸 | 🗸 | 🗸 | 🗸 | 🗸 | 🗸 | 🗸 | 🗸 | 🗸 | 🗸 | **-** |
| *Ptaeroxylon obliquum* | 🗸 | 🗸 | 🗸 | 🗸 | 🗸 | 🗸 | 🗸 | 🗸 | **-** | **-** | 🗸 | 🗸 | 🗸 | 🗸 |
| *Scutia myrtina* | 🗸 | 🗸 | **-** | **-** | 🗸 | 🗸 | **-** | 🗸 | **-** | **-** | **-** | **-** | 🗸 | 🗸 |
| *Cassipourea gummiflua*^V^ | **-** | 🗸 | **-** | **-** | **-** | **-** | **-** | **-** | **-** | **-** | **-** | **-** | **-** | **-** |
| *Cassipourea malosana*^D^ | 🗸 | 🗸 | **-** | **-** | **-** | **-** | 🗸 | **-** | **-** | **-** | **-** | **-** | **-** | **-** |
| *Prunus africana*^V^ | 🗸 | 🗸 | **-** | **-** | **-** | **-** | **-** | **-** | **-** | **-** | **-** | **-** | 🗸 | **-** |
| *Canthium ciliatum* | **-** | 🗸 | **-** | **-** | **-** | **-** | **-** | **-** | **-** | **-** | **-** | 🗸 | 🗸 | **-** |
| *Canthium kuntzeanum* | **-** | **-** | **-** | **-** | **-** | **-** | **-** | **-** | **-** | **-** | 🗸 | **-** | **-** | 🗸 |
| *Hypecanthus amoenus* | 🗸 | 🗸 | 🗸 | 🗸 | **-** | **-** | **-** | **-** | **-** | **-** | **-** | 🗸 | **-** | 🗸 |
| *Tricalysia lanceolata*^E^ | **-** | 🗸 | 🗸 | 🗸 | **-** | **-** | **-** | **-** | **-** | **-** | **-** | **-** | **-** | **-** |
| *Calodendrum capense* | 🗸 | 🗸 | 🗸 | 🗸 | 🗸 | 🗸 | 🗸 | 🗸 | **-** | **-** | **-** | **-** | 🗸 | 🗸 |
| *Clausena anisata* | 🗸 | 🗸 | 🗸 | 🗸 | **-** | **-** | 🗸 | **-** | **-** | **-** | 🗸 | **-** | 🗸 | 🗸 |
| *Vepris lanceolata* | **-** | 🗸 | 🗸 | 🗸 | **-** | **-** | **-** | **-** | **-** | **-** | **-** | **-** | **-** | **-** |
| *Zanthoxylum capense* | 🗸 | 🗸 | 🗸 | 🗸 | **-** | **-** | **-** | 🗸 | **-** | **-** | 🗸 | 🗸 | 🗸 | 🗸 |
| *Zanthoxylum dayvi* | 🗸 | 🗸 | **-** | **-** | **-** | **-** | 🗸 | **-** | **-** | **-** | **-** | **-** | 🗸 | 🗸 |
| *Allophylus africanus* | 🗸 | 🗸 | **-** | **-** | **-** | **-** | **-** | **-** | **-** | **-** | **-** | **-** | 🗸 | **-** |
| *Allophylus dregeanus* | **-** | 🗸 | **-** | **-** | **-** | **-** | **-** | **-** | **-** | **-** | **-** | **-** | 🗸 | **-** |
| *Halleria lucida* | 🗸 | 🗸 | 🗸 | 🗸 | 🗸 | 🗸 | 🗸 | 🗸 | 🗸 | 🗸 | 🗸 | **-** | 🗸 | 🗸 |
| *Solanum giganteum* | **-** | 🗸 | **-** | 🗸 | **-** | **-** | **-** | **-** | **-** | **-** | **-** | **-** | **-** | **-** |
| **Solanum mauritianum* | 🗸 | 🗸 | 🗸 | 🗸 | 🗸 | 🗸 | 🗸 | 🗸 | **-** | **-** | **-** | 🗸 | 🗸 | 🗸 |
| *Dais cotinifolia* | 🗸 | 🗸 | **-** | **-** | **-** | **-** | **-** | **-** | **-** | **-** | **-** | **-** | 🗸 | **-** |
| *Peddiea africana* | **-** | **-** | **-** | **-** | 🗸 | **-** | **-** | **-** | **-** | **-** | **-** | **-** | **-** | **-** |
| Species richness | **52** | **59** | **33** | **32** | **24** | **25** | **27** | **18** | **7** | **5** | **30** | **22** | **41** | **30** |

1= Karkloof Nature Reserve, 2 = Mbona Private Nature Reserve, 3 = L’Abri, 4= Benvie Farm, 5 = Rameron, 6 = Milestone Forest Walk, 7 = Maritzdaal, 8 = Sharedown Forest, 9 = Wakefield Forest, 10=Waterfall Forest, 11 = Ingelabantwana Nature Reserve, 12 = Xotsheyake Nature Reserve, 13 = Marutswa Nature Reserve, 14 = Nxumeni Forest

*Alien invasive

^D^ Declining

^E^ Endangered

^P^ Protected

^V^ Vulnerable

**Supplementary Information Table S5** Bird species recorded and seasonal counts in 14 selected Mistbelt forest patches of the Midlands of KwaZulu-Natal, South Africa

| **Family** | **Scientific name** | **Breeding season (n)** | **Non-breeding season (n)** |
| --- | --- | --- | --- |
| Accipitridae | *Buteo trizonatus*^NT^ | 10 | 0 |
|  | *Lophaetus occipitalis* | 16 | 27 |
|  | *Polemaetus bellicosus*^E;V^ | 0 | 3 |
|  | *Stephanoaetus coronatus* | 5 | 0 |
| Alcedinidae | *Ispidina picta* | 3 | 0 |
| Bucerotidae | *Bycanistes bunicator* | 28 | 7 |
|  | *Tockus alboteerminatus*^V;NT^ | 4 | 8 |
| Bucorvidae | *Bucorvus leadbeateri*^E;V^ | 10 | 14 |
| Campephagidae | *Campephaga flava* | 8 | 7 |
|  | *Coracina caesia* | 89 | 71 |
| Cisticolidae | *Apalis flavida* | 37 | 7 |
|  | *Apalis thoracica* | 487 | 328 |
|  | *Camaroptera brachyura* | 423 | 253 |
|  | *Cisticola aberrans* | 2 | 0 |
|  | *Prinia subflava* | 46 | 61 |
| Coliidae | *Colius striatus* | 18 | 48 |
| Columbidae | *Aplopelia larvata* | 79 | 75 |
|  | *Columba arquatrix* | 231 | 117 |
|  | *Columba delegorguei*^E^ | 5 | 0 |
|  | *Streptopelia capicola* | 51 | 38 |
|  | *Streptopelia semitorquata* | 96 | 124 |
|  | *Streptopelia senegalensis* | 4 | 2 |
|  | *Treron calvus* | 2 | 0 |
|  | *Turtur tympanistria* | 12 | 6 |
| Cuculidae | *Centropus burchelliii* | 2 | 0 |
|  | *Chrysococcyx capreus* | 90 | 1 |
|  | *Chrysococcyx caprius* | 13 | 0 |
|  | *Chrysococcyx klaas* | 2 | 0 |
|  | *Clamatar jacobinus* | 7 | 0 |
|  | *Dicrurus adsimilis* | 91 | 73 |
|  | *Cuculus clamosus* | 41 | 0 |
|  | *Cuculus solitarius* | 196 | 0 |
| Estreldidae | *Coccopygia melanotis* | 1 | 1 |
|  | *Estrilda astrild* | 0 | 24 |
|  | *Lagonostricta rhodopareia* | 39 | 246 |
|  | *Spermestes cucullata* | 6 | 3 |
| Fringillidae | *Crithagra scotops* | 30 | 16 |
|  | *Crithagra sulphurata* | 3 | 0 |
| Indicatoridae | *Indicator minor* | 7 | 0 |
|  | *Indicator variegatus* | 13 | 0 |
| Laniidae | *Lanius collaris* | 0 | 1 |
| Locustellidae | *Bradypterus barratti* | 20 | 28 |
| Lybiidae | *Pogoniulus pusillus* | 1 | 0 |
|  | *Lybius torquatus* | 0 | 1 |
| Macrosphenidae | *Sylvietta rufescens* | 1 | 0 |
| Malaconotidae | *Dryoscopus cubla* | 84 | 11 |
|  | *Laniarius ferrugineus* | 657 | 656 |
|  | *Malaconotus blanchoti* | 3 | 0 |
|  | *Telophorus olivaceus* | 86 | 70 |
|  | *Telophorus viridis* | 2 | 0 |
| Mornachidae | *Trochocercus cyanomelas* | 80 | 55 |
|  | *Terpsiphone viridis* | 44 | 12 |
| Muscicapidae | *Cercotrichas leucophrys* | 5 | 5 |
|  | *Cossypha caffra* | 86 | 113 |
|  | *Cossypha dichroa* | 144 | 10 |
|  | *Cossypha natalensis* | 8 | 14 |
|  | *Melaenornis pammelaina* | 1 | 0 |
|  | *Muscicapa adusta* | 46 | 10 |
|  | *Muscicapa caerulescens* | 6 | 0 |
|  | *Myioparus plumbeus* | 5 | 0 |
|  | *Pogonocichla stellata* | 76 | 10 |
|  | *Tauraco corythaix* | 183 | 245 |
| Oriolidae | *Orious larvatus* | 190 | 73 |
| Nectariniidae | *Cinnyris chalybeus* | 271 | 538 |
|  | *Chalcomitra amethystina* | 87 | 5 |
|  | *Cyanomitra olivacea* | 22 | 0 |
|  | *Hedysdipna collaris* | 153 | 47 |
|  | *Nectarinia fanosa* | 9 | 0 |
| Numididae | *Guttera edouardii* | 103 | 67 |
|  | *Numida meleagris* | 25 | 10 |
| Paridae | *Parus niger* | 3 | 0 |
| Phasianidae | *Pternistis natalensis* | 5 | 0 |
|  | *Pternitsis afer* | 28 | 16 |
|  | *Pternitsis swainsonii* | 17 | 0 |
| Phoeniculidae | *Phoeniculus purpureus* | 24 | 8 |
| Phylloscopidae | *Phylloscopus ruficapilla* | 102 | 0 |
| Picidae | *Campethera abingoni* | 7 | 0 |
|  | *Campethera notata*^NT^ | 4 | 46 |
|  | *Dendropicos griseocephalus* | 56 | 78 |
| Platysteiridae | *Batis capensis* | 191 | 140 |
| Ploceidae | *Ploceous bicolor* | 65 | 40 |
|  | *Ploceous ocularis* | 1 | 3 |
|  | *Ploceus velatus* | 1 | 2 |
| Psittacidae | *Poicephalus robustus*^E;V^ | 40 | 95 |
| Pycnonotidae | *Andropadus importunus* | 557 | 327 |
|  | *Phyllastrephus terrestris* | 192 | 374 |
|  | *Pycnonotus tricolor* | 127 | 475 |
| Sarothruridae | *Sarothruta elegans* | 49 | 7 |
| Slyviidae | *Lioptilus nigricapillus*^V^ | 14 | 12 |
| Strurndiae | *Onychognathus morio* | 0 | 27 |
| Threskiornithidae | *Bostrychia hagedash* | 284 | 316 |
| Trogonidae | *Apaloderma narina* | 84 | 6 |
| Turdidae | *Turdus olivaceous* | 42 | 20 |
|  | *Geokichla gurneyi*^NT^ | 57 | 50 |
| Upupidae | *Upupa africana* | 4 | 0 |
| Zosteropidae | *Zosterops virens* | 373 | 420 |

^E^ Endangered

^NT^ Near-threatened

^V^ Vulnerable

**Supplementary Information Table S6.** Indicator bird species for selected Southern Mistbelt forest patches of sizes 200-500 and >500 ha in the Midlands of KwaZulu-Natal, South Africa.

| **Forest patch size (ha)** | **Bird species** | **Habitat specificity** | **Stat** | **p-value** |
| --- | --- | --- | --- | --- |
| >200-500 | *Bucorvus leadbeateri* | Generalist | 0.827 | 0.04 |
| >500 | *Trochocercus cyanomela* | Specialist | 0.930 | 0.001 |
|  | *Prinia subflava* | Generalist | 0.926 | 0.001 |
|  | *Tauraco corythaix* | Specialist | 0.918 | 0.001 |
|  | *Pycnonotus tricolor* | Generalist | 0.910 | 0.01 |
|  | *Cossypha caffra* | Generalist | 0.906 | 0.01 |
|  | *Phyllastrephus terrestis* | Generalist | 0.905 | 0.01 |
|  | *Cinnyris chalbeus* | Generalist | 0.899 | 0.001 |
|  | *Ploceus bicolor* | Specialist | 0.893 | 0.001 |
|  | *Apalis flavida* | Generalist | 0.888 | 0.01 |
|  | *Camaroptera brachyura* | Specialist | 0.875 | 0.01 |
|  | *Laniarius ferrugineus* | Generalist | 0.870 | 0.01 |
|  | *Batis capensis* | Specialist | 0.869 | 0.01 |
|  | *Chlorophoneus olivaceus* | Specialist | 0.865 | 0.001 |
|  | *Andropadus importunus* | Generalist | 0.858 | 0.01 |
|  | *Dendropicos griseocephalus* | Specialist | 0.855 | 0.001 |
|  | *Cossphya natalensis* | Specialist | 0.850 | 0.01 |
|  | *Bradypterus barratti* | Generalist | 0.837 | 0.02 |
|  | *Apalis thoracica* | Specialist | 0.833 | 0.01 |
|  | *Lophaetus occipitalis* | Generalist | 0.820 | 0.01 |
|  | *Chalcomitra amethystina* | Generalist | 0.819 | 0.01 |
|  | *Zosterops capensis* | Generalist | 0.817 | 0.01 |
|  | *Oriolus larvatus* | Generalist | 0.813 | 0.01 |
|  | *Coracina caesia* | Specialist | 0.810 | 0.001 |
|  | *Hedypina collaris* | Specialist | 0.804 | 0.01 |
|  | *Chrysococyx cupreus* | Specialist | 0.784 | 0.01 |
|  | *Upupa africana* | Generalist | 0.775 | 0.04 |
|  | *Columba larvata* | Specialist | 0.767 | 0.01 |
|  | *Pogonocichla stellata* | Specialist | 0.759 | 0.01 |
|  | *Dicrucus admilis* | Generalist | 0.755 | 0.03 |
|  | *Cuculus solitarius* | Specialist | 0.748 | 0.01 |
|  | *Apaloderma narina* | Specialist | 0.735 | 0.02 |
|  | *Cyanomitra olivacea* | Specialist | 0.732 | 0.03 |
|  | *Campephaga flava* | Generalist | 0.721 | 0.04 |
|  | *Geokichla gurneyi* | Specialist | 0.718 | 0.04 |
|  | *Dryoscopus cubla* | Generalist | 0.718 | 0.02 |
|  | *Cossypha dichroa* | Specialist | 0.702 | 0.03 |
|  | *Terpsiphone viridis* | Specialist | 0.687 | 0.03 |
|  | *Chrysococyx caprius* | Generalist | 0.668 | 0.04 |
|  | *Sarothrura elegans* | Specialist | 0.663 | 0.04 |
|  | *Phylloscopus ruficapilla* | Specialist | 0.661 | 0.03 |

| **Non-breeding**  **Supplementary Information Table S7.** Modelled-averaged coefficients for the GLM top models of the effects of tree species richness (TRic), structural complexity (SC), canopy cover (CC), water cover (WC) and leaf litter (LL) on bird species richness and functional diversity (Fric, FEve, FDiv) of avian communities of the Midlands Mistbelts forest patches in the breeding and non-breeding season in KwaZulu-Natal, South Africa. |  |  |  |  |  |
| --- | --- | --- | --- | --- | --- |
| **Response** | **Variable** | **Estimate** | **Std error** | **z-value** | **p-value** |
| Bird species richness | Tree species richness | **0.165** | **0.056** | **2.985** | **0.0028** |
|  | Structural complexity | **0.286** | **0.064** | **5.667** | **5.16e-06** |
|  | Canopy cover | -0.064 | 0.021 | 1.092 | 0.232 |
|  | Water cover | 0.014 | 0.004 | 0.383 | 0.651 |
| FRic (All) | Structural complexity | 0.163 | 0.012 | 1.434 | 0.155 |
|  | Tree species richness | 0.142 | 0.010 | 1.300 | 0.114 |
|  | Canopy cover | -0.053 | 0.011 | 0.839 | 0.394 |
| FRic (Specialists) | Tree species richness | **0.245** | **0.173** | **1.376** | **0.019** |
|  | Structural complexity | **0.523** | **0.290** | **1.653** | **0.036** |
| Fric (Generalists) | Structural complexity | 0.264 | 0.192 | 1.384 | 0.178 |
|  | Canopy cover | -0.246 | 0.169 | 1.438 | 0.155 |
|  | Tree species richness | 0.1883 | 0.1629 | 1.165 | 0.260 |
|  | Water cover | -0.047 | 0.143 | 0.328 | 0.743 |
| FEve (All) | Structural complexity | -0.144 | 0.048 | 0.354 | 0.726 |
|  | Tree species richness | 0.119 | 0.052 | 0.143 | 0.882 |
|  | Canopy cover | 0.081 | 0.044 | 0.120 | 0.867 |
|  | Water cover | -0.033 | 0.045 | 0.071 | 0.948 |
| FEve (Specialists) | Structural complexity | **0.596** | **0.299** | **0.102** | **0.014** |
|  | Tree species richness | 0.157 | 0.055 | 0.342 | 0.730 |
|  | Water cover | 1.295 | 0.126 | 0.173 | 0.863 |
|  | Canopy cover | -0.115 | 0.046 | 0.249 | 0.804 |
| FEve (Generalists) | Canopy cover | **-0.152** | **0.041** | **0.126** | **0.008** |
|  | Structural complexity | -0.102 | 0.038 | 0.265 | 0.790 |
|  | Tree species richness | -0.166 | 0.424 | 0.392 | 0.695 |
|  | Water cover | -1.819 | 0.206 | 0.093 | 0.926 |
| FDiv (All) | Tree species richness | -0.038 | 0.304 | 0.124 | 0.901 |
| FDiv (Specialists) | Structural complexity | **0.403** | **0.286** | **0.610** | **0.042** |
|  | Tree species richness | 0.150 | 0.130 | 0.401 | 0.313 |
|  | Canopy Cover | -0.125 | 0.030 | 0.210 | 0.068 |
|  | Water cover | -2.625 | 1.463 | 0.351 | 0.844 |
| FDiv (Generalists) | Structural complexity | 0.223 | 0.166 | 0.544 | 0.654 |
|  | Canopy Cover | -**0.231** | **0.131** | **0.519** | **0.001** |
|  | Water cover | 0.035 | 0.037 | 0.153 | 0.944 |
|  | Tree species richness | 0.135 | 0.091 | 0.274 | 0.788 |
| Breeding |  |  |  |  |  |
| Response | Variable |  |  |  |  |
| Bird species richness | Tree species richness | **0.261** | **0.052** | **4.337** | **4.24e-05** |
|  | Canopy cover | 0.079 | 0.063 | 1.264 | 0.226 |
|  | Leaf Litter | 0.031 | 0.067 | 0.413 | 0.737 |
|  | Structural complexity | 0.052 | 0.035 | 0.733 | 0.435 |
| Fric(All) | Tree species richness | 0.179 | 0.108 | 0.727 | 0.467 |
|  | Leaf Litter | -0.016 | 0.010 | 0.150 | 0.882 |
|  | Canopy cover | 0.115 | 0.104 | 0.148 | 0.882 |
|  | Structural complexity | 0.121 | 0.111 | 0.171 | 0.866 |
| Forest specialists | Tree species richness | 0.167 | 0.137 | 1.385 | 0.190 |
|  | Structural complexity | -0.177 | 0.144 | 0.534 | 0.594 |
|  | Canopy cover | -0.109 | 0.130 | 0.070 | 0.944 |
|  | Leaf litter | -0.501 | 0.133 | 0.513 | 0.619 |
| Forest generalists | Tree species richness | 0.153 | 0.134 | 0.740 | 0.256 |
|  | Canopy cover | 0.276 | 0.212 | 0.528 | 0.598 |
|  | Leaf litter | -0.044 | 1.343 | 0.291 | 0.777 |
|  | Structural complexity | -0.141 | 0.140 | 0.270 | 0.793 |
| FEve (All) | Tree species richness | 0.139 | 0.447 | 0.311 | 0.756 |
|  | Canopy cover | 0.167 | 0.107 | 0.040 | 0.968 |
|  | Leaf Litter | 0.135 | 0.132 | 0.086 | 0.932 |
|  | Structural complexity | -0.133 | 0.160 | 0.082 | 0.935 |
| Forest specialists | Tree species richness | **0.417** | **0.008** | **0.024** | **0.004** |
|  | Leaf litter | 0.041 | 0.015 | 0.090 | 0.999 |
|  | Canopy cover | 0.101 | 0.015 | 0.002 | 0.929 |
|  | Structural complexity | 0.109 | 0.130 | 0.024 | 0.981 |
| Forest generalists | Structural complexity | **0.216** | **0.031** | **0.135** | **0.001** |
|  | Tree species richness | 0.112 | 0.135 | 0.054 | 0.210 |
|  | Leaf litter | 0.123 | 0.021 | 0.192 | 0.801 |
|  | Canopy cover | 2.124 | 1.114 | 0.168 | 0.621 |
| FDiv (All) | Tree species richness | 1.014 | 0.320 | 0.044 | 0.965 |
|  | Structural complexity | -1.014 | 0.302 | 0.042 | 0.966 |
|  | Canopy cover | -1.010 | 0.308 | 0.034 | 0.972 |
|  | Leaf Litter | -1.015 | 0.321 | 0.045 | 0.964 |
| Forest specialists | Tree species richness | **0.160** | **0.029** | **0.054** | **0.002** |
|  | Leaf litter | -1.010 | 0.293 | 0.036 | 0.971 |
|  | Canopy cover | -1.002 | 0.293 | 0.005 | 0.996 |
|  | Structural complexity | **0.150** | **0.022** | **0.052** | **0.010** |
| Forest generalists | Tree species richness | 0.036 | 0.003 | 0.112 | 0.923 |
|  | Structural complexity | **0.344** | **0.041** | **0.129** | **0.001** |
|  | Canopy cover | 1.085 | 0.355 | 0.243 | 0.808 |
|  | Leaf litter | -1.023 | 0.335 | 0.073 | 0.941 |
